# A Systems-Level Transcriptomic Framework Identifies Shared Cellular Hubs in Osteoarthritis and Alzheimer’s Disease

**DOI:** 10.34133/csbj.0085

**Published:** 2026-05-13

**Authors:** Zhangzheng Wang, Krisztián Juhász Zoltán, Csaba Matta, Luca Paluska, Ahmed Al-Mnaseer, Roland Takács, László Ducza

**Affiliations:** ^1^ Department of Anatomy, Histology and Embryology, Faculty of Medicine, University of Debrecen, H-4032 Debrecen, Hungary.; ^2^ Department of Biomedical Materials Science, Graduate School of JABA, Wonkwang University, Iksan, Republic of Korea.; ^3^ Integrated Omics Institute, Wonkwang University, Iksan, Republic of Korea.

## Abstract

Osteoarthritis (OA) and Alzheimer’s disease (AD) are prevalent age-associated disorders that frequently co-occur, yet the molecular basis of their comorbidity remains incompletely understood. To explore potential shared cellular programs, we performed an integrative analysis of publicly available bulk and single-cell transcriptomic datasets derived from human OA cartilage and AD cortex. Cross-disease comparison identified 60 overlapping differentially expressed genes, including 18 consistently up-regulated genes, which we defined as a shared up-regulated gene set (SUGS). Functional enrichment analyses indicated convergence on extracellular matrix remodeling, inflammatory signaling, metabolic stress responses, and immune regulation. Single-cell analysis of OA cartilage revealed expansion of a fibrochondrocyte subpopulation enriched for SUGS activity and extracellular-matrix-associated ligands. In AD cortex, a disease-associated oligodendrocyte subcluster displayed elevated SUGS activity and stress-response gene expression and occupied a prominent-receiver-like position within inferred neuronal–glial communication networks. Ligand–receptor analysis performed independently within each tissue identified collagen-related signaling in OA and neurexin-associated signaling in AD as dominant intratissue pathways. Because the OA and AD datasets are cross-sectional and were derived from independent cohorts and distinct tissues, these analyses do not establish direct inter-organ communication, temporal sequence, or causal directionality. In addition, CellChat-based ligand–receptor inference was performed independently within each tissue and does not itself infer cross-organ communication. The identification of sender-like and receiver-like cellular hubs should therefore be interpreted as a hypothesis-generating, systems-level conceptual framework that may help organize future experimental studies investigating potential links between joint inflammation and neurodegeneration in aging.

## Highlights

• Integrated bulk and single-cell transcriptomics identified potentially shared osteoarthritis (OA)–Alzheimer’s disease (AD) molecular programs.
• An 18-gene shared up-regulated gene set (SUGS) was detected across OA and AD.
• Fibrochondrocyte_1 emerged as a dominant-sender-like hub in OA cartilage.
• Oligodendrocyte_3 emerged as a prominent-receiver-like hub in AD cortex.
• Findings support a hypothesis-generating systems framework for OA–AD comorbidity.

## Graphical Abstract

**Figure:**
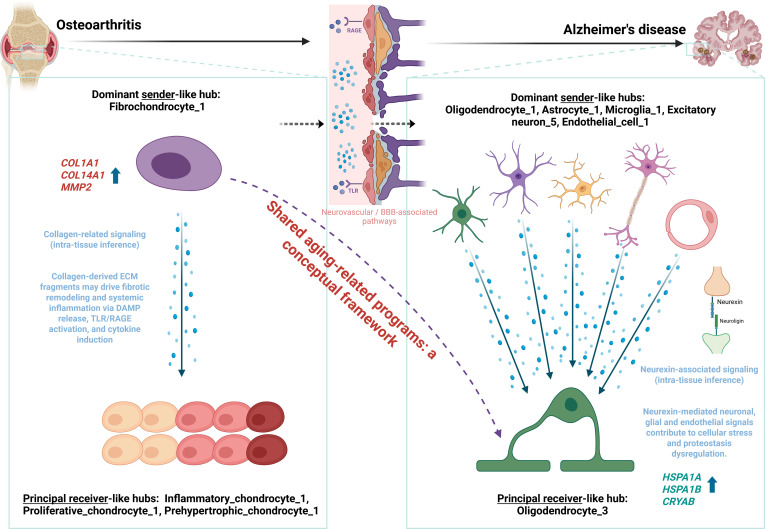

## Introduction

Osteoarthritis (OA) and Alzheimer’s disease (AD) are 2 of the most prevalent and disabling age-associated disorders, each imposing substantial and increasing global health and socioeconomic burdens. Traditionally, OA and AD have been considered etiologically distinct conditions, primarily affecting the musculoskeletal and central nervous system, respectively. However, accumulating epidemiological and molecular evidence suggests that these disorders may share overlapping aging-related biological features [1]. Emerging literature has introduced the concept of a “brain–joint axis”, describing potential neuroimmune and systemic interactions that could link peripheral joint pathology with central nervous system dysfunction [2]. While this framework provides a useful conceptual model, the extent to which OA and AD are mechanistically connected, as opposed to reflecting parallel manifestations of aging-associated inflammation and metabolic dysregulation, remains incompletely understood.

OA is the most common degenerative joint disorder, affecting over 600 million people globally, with incidence projected to rise substantially by 2050 [3]. Once regarded primarily as a mechanical “wear-and-tear” condition, OA is now recognized as a multifactorial whole-joint disease characterized by progressive cartilage degradation, subchondral bone remodeling, synovial inflammation, and chronic pain [4,5]. Chronic low-grade inflammation and extracellular matrix (ECM) remodeling are central components of OA pathophysiology, with inflammatory mediators and matrix fragments contributing to sustained local tissue stress [6].

AD, the leading cause of dementia, currently affects approximately 50 million individuals worldwide, with projections exceeding 150 million cases by 2050 [7]. It is characterized by extracellular amyloid-β deposition, intracellular hyperphosphorylated tau accumulation, synaptic dysfunction, metabolic stress, and chronic neuroinflammation [8–11]. Increasing evidence highlights the role of immune dysregulation, oxidative stress, and mitochondrial dysfunction in AD progression, particularly in aging populations.

Central to both OA and AD is the disruption of tissue homeostasis associated with aging [6,12]. Chronic low-grade systemic inflammation has been proposed as a shared feature linking peripheral and central pathology [2]. In OA, inflammatory mediators including cytokines and matrix-derived fragments may enter systemic circulation. Experimental studies suggest that under certain conditions, systemic inflammation can influence blood–brain barrier (BBB) integrity and microglial activation [13–15]. However, whether such mechanisms directly mediate OA–AD comorbidity in humans remains unresolved. These findings collectively support the possibility of neuroimmune cross-talk, although causal directionality has yet to be firmly established.

Clinical and epidemiological observations further support an association between OA and cognitive decline. In mouse models of AD, peripheral joint inflammation has been reported to exacerbate amyloid-β pathology and glial activation [16]. In older adults without dementia, OA has been associated with accelerated hippocampal atrophy [17], and population-based studies link OA and chronic pain burden to increased dementia risk [18–20]. While these findings suggest a relationship between joint pathology and neurodegeneration, they do not establish direct mechanistic communication and may reflect shared systemic risk factors.

At the molecular level, transcriptomic studies have identified overlapping dysregulated genes and signaling pathways in OA and AD, including inflammatory mediators, ECM-associated proteins, and regulators of metabolic and stress responses [21]. Recent pathway-oriented computational analyses have further emphasized the value of systems-level approaches for identifying AD-related biological programs [22]. Convergent pathways such as nuclear factor κB activation, phosphatidylinositol 3-kinase–Akt signaling, mitochondrial dysfunction, and autophagy regulation have been implicated in both diseases [2]. Metabolic comorbidities, including obesity and insulin resistance, further compound risk and progression under both OA and neurodegenerative conditions [2]. Nevertheless, bulk transcriptomic approaches lack cell type resolution, limiting insight into how specific cellular populations may contribute to these shared programs.

Despite accumulating evidence of overlapping pathways, causality and directionality remain difficult to establish because of cellular heterogeneity and the use of independent cohorts across studies. Furthermore, Mendelian randomization analyses have yielded mixed results regarding direct causal links [23]. To address these limitations, we performed an integrative cross-disease analysis combining bulk and single-cell RNA sequencing (RNA-seq) datasets from human OA cartilage and AD cortex. Our objective was not to presuppose direct inter-organ signaling but rather to identify shared molecular signatures and cell-type-specific transcriptional programs that may provide a systems-level framework for understanding OA–AD comorbidity. By integrating differential expression, pathway enrichment, and intratissue intercellular communication analyses, we sought to generate testable hypotheses regarding potential cellular hubs that could contribute to parallel inflammatory and stress-response mechanisms in aging.

## Materials and Methods

**Fig. 1.**
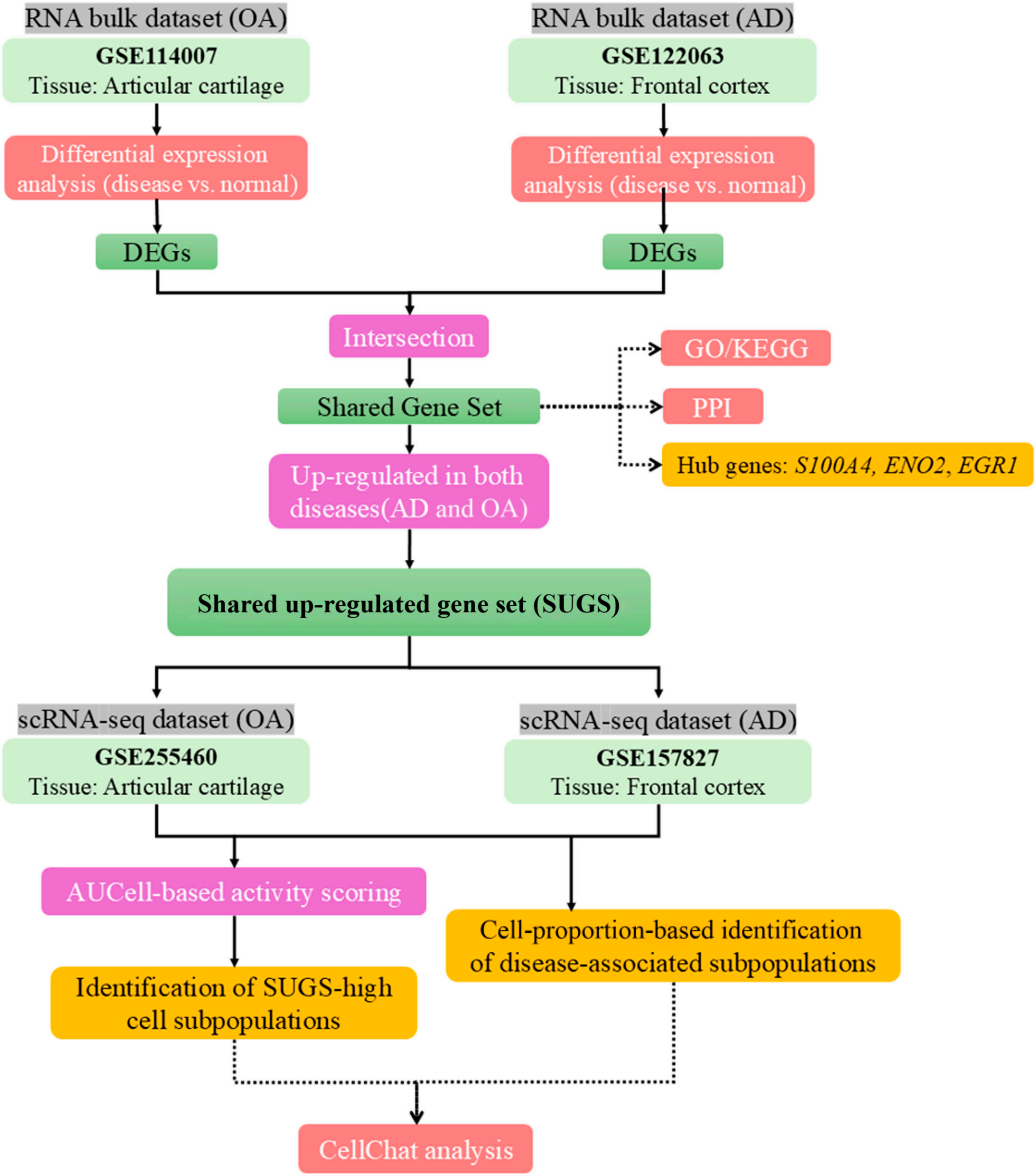
Integrative workflow combining bulk and single-cell transcriptomic data from osteoarthritis (OA) and Alzheimer’s disease (AD). Shared up-regulated gene sets (SUGSs) were identified from bulk RNA sequencing (RNA-seq) and microarray datasets (GSE114007: OA cartilage [bulk RNA-seq]; GSE122063: AD cortex [microarray dataset] and projected onto matching single-cell datasets; GSE255460: OA knee cartilage scRNA-seq; GSE157827: AD cortex scRNA-seq) for cell type localization. SUGS activity was assessed via AUCell scoring, with ligand–receptor interactions inferred using CellChat to identify key sender–receiver populations.

This study adopted a signature-based integration strategy to align bulk-identified disease signals with single-cell-resolved cellular contexts across OA and AD. We assume that a shared gene set reflects conserved, cell-intrinsic programs contributing to both diseases. Differential expression analysis (disease versus normal) was performed separately on OA and AD bulk datasets to obtain differentially expressed genes (DEGs), and the intersection of these DEGs was defined as the shared gene set. Genes up-regulated in both diseases were retained as the shared up-regulated gene set (SUGS). SUGS was then projected onto the corresponding single-cell datasets to localize their activity by (a) computing per-cell enrichment scores (AUCell) and (b) comparing activity across annotated clusters to identify cell types preferentially carrying the signature. These SUGS-high populations were further contextualized with pathway enrichment and ligand–receptor interaction modeling. Tissue selection was restricted to cartilage (OA) and cortex (AD) to ensure direct disease relevance (Fig. 1).

### Data collection

Bulk RNA-seq data for OA were obtained from the publicly available GSE114007 dataset [24], which was generated on the Illumina HiSeq 2000 (*Homo sapiens*) platform (GPL11154) and includes articular cartilage samples from 8 healthy donors and 10 patients with OA. Corresponding transcriptomic data for AD were retrieved from the microarray dataset GSE122063 [25], which was generated on the Agilent-039494 SurePrint G3 Human GE v2 8x60K microarray platform (GPL16699) and includes frontal cortex tissue samples from 24 cognitively normal controls and 28 patients with AD. Prior to differential expression analysis, low-quality or low-abundance genes were filtered out in both datasets to reduce noise. For the microarray-derived AD dataset, probe-to-gene mapping was performed using the GPL16699 platform annotation, and when multiple probes mapped to the same gene symbol, we summarized probe-level intensities by taking their mean as the gene-level expression.

Single-cell RNA-seq (scRNA-seq) datasets were compiled to achieve cell-type-specific resolution of gene expression changes. scRNA-seq data for OA were accessed from GSE255460 [26], including 19 knee cartilage samples from 8 patients with OA and 3 healthy control donors. For AD, scRNA-seq data were extracted from the GSE157827 dataset [27], including 21 prefrontal cortex tissue samples from 12 patients with AD and 9 cognitively healthy controls (Data File S1). An integrative analytical workflow was adopted to combine bulk and single-cell transcriptomic data, enabling comprehensive cross-disease and cross-tissue comparisons. This approach facilitates the identification of shared molecular programs and putative intercellular communication networks implicated in OA and AD pathogenesis. The overall workflow, including data acquisition, preprocessing, integration, and downstream analyses, is illustrated in Fig. 1.

### Differential expression analysis

Differential expression pipelines were tailored to the specific platform to maximize statistical accuracy and biological relevance. For the bulk RNA-seq data obtained from OA articular cartilage samples, the DESeq2 package (version 1.50.2) was applied [28], taking advantage of its negative binomial model suited for count-based data. For the microarray-derived AD dataset, the limma package (version 3.64.3) was used [29], applying linear modeling and empirical Bayes moderation appropriate for expression array data.

Genes with adjusted *P* values (Benjamini–Hochberg [BH] correction) below 0.05 and log_2_ fold change (|log_2_FC|) ≥ 1 were considered DEGs, ensuring statistical stringency while capturing biologically relevant transcriptional changes. Overlap significance was quantified using a one-sided hypergeometric test (phyper function). Cross-dataset effect-size concordance was assessed using Spearman’s rank correlation (cor.test function).

Functional enrichment analyses of shared DEGs were performed using the clusterProfiler package (version 4.16.0) to identify key biological processes, pathways, and molecular functions package [30]. Adjusted *P* values were calculated using the BH method to control the false discovery rate (FDR). Subsequently, the combined list of shared DEGs was uploaded into the STRING database (https://cn.string-db.org/) with species parameter set to *Homo sapiens* and minimum interaction confidence to 0.4. The resulting protein–protein interaction (PPI) network was visualized and analyzed using Cytoscape (v.3.8.0) to identify key molecular hubs and network modules critical for shared pathophysiology [31].

### Single-cell data analysis

Principal components analysis (PCA) was applied for dimensionality reduction, enabling identification of the primary axes of transcriptional variation prior to downstream analyses. For focused biological interpretation, we concentrated on overlapping up-regulated DEGs shared between OA and AD, termed SUGS. For all single-cell datasets, samples were first evaluated based on post-quality-control cell yields. Samples retaining fewer than 4,000 high-quality cells after quality filtering were excluded from downstream analyses.

For the OA scRNA-seq dataset, 2 samples (C1 and OA7_1) were excluded because of insufficient cell counts. Cells were filtered on the basis of quality metric retaining those with ≥500 unique molecular identifiers, between 200 and 4,000 detected genes, novelty score of >0.8, and <10% mitochondrial gene content. Data normalization, variable feature selection (top 2,000 genes), and scaling were performed using Seurat’s NormalizeData, FindVariableFeatures, and ScaleData functions, respectively [32]. PCA was performed with RunPCA, and Harmony integration [32] was applied using 30 PCs and a resolution of 0.4 to correct for batch effects, followed by clustering at a resolution of 0.7.

For the AD scRNA-seq dataset, 4 AD samples (AD6, AD8, AD13, and AD19) and 2 normal control samples (NC11 and NC16) were excluded because of quality concerns. Cells were retained if they had total unique molecular identifiers below 20,000, detected genes above 200, and mitochondrial reads under 20%. Normalization, feature selection, scaling, PCA, and batch effect correction using Harmony were performed as above but with 20 PCs and a resolution of 0.4. Cluster-specific DEGs were identified using Seurat’s FindAllMarkers function with a log_2_FC threshold of 0.25, using Wilcoxon rank-sum testing (test.use = “wilcox”).

As the primary purpose of this study was to establish a consistent cross-disease integration framework, Harmony was applied with uniform parameterization across platforms; alternative integration strategies (e.g., Seurat v3 anchoring) were not systematically evaluated. Cellular populations were identified and annotated using a marker-based strategy guided by the original source publications for each dataset. SUGS pathway activity per cell was quantified with AUCell (version 1.30.1) [33] and visualized using ggplot2 (version 4.0.2). Cell–cell communication networks were inferred using CellChat (version 1.6.1) [34] using only the cells from the disease group. CellChat-based ligand–receptor interactions are inferred from gene expression patterns and curated databases and do not constitute direct evidence of functional signaling.

Cells from all disease and control samples were jointly processed and clustered to ensure consistent cell type definitions across conditions. To assess shifts in cellular composition, differential abundance was evaluated at the donor level by computing the proportion of each cell type per donor and comparing disease versus control groups using Wilcoxon rank-sum tests with FDR correction (BH). To validate transcriptional alterations independent of single-cell variability, raw counts for each (cell type × donor) were aggregated to generate pseudobulk profiles, and differential expression between groups was tested using the edgeR quasi-likelihood framework, with FDR controlled by the default BH method.

Single-cell data analysis was performed using the Seurat (V5) R package (version 5.4.0), and all computational analyses were carried out in R version 4.5.1.

## Results

### Shared gene expression profiles and pathway dysregulation across OA and AD tissues

**Fig. 2.**
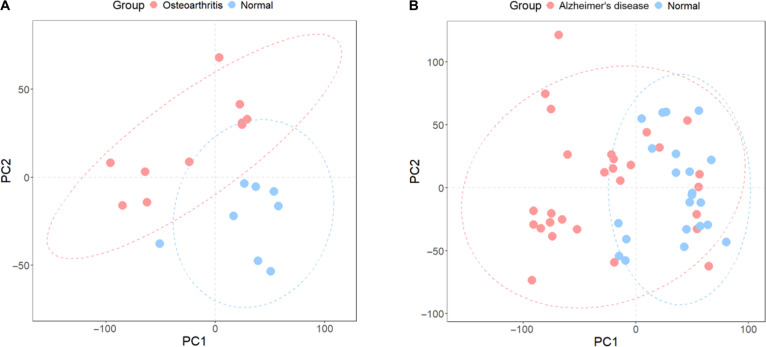
Principal components analysis (PCA) of bulk RNA sequencing (RNA-seq) and microarray datasets. (A) Osteoarthritis (OA) bulk RNA-seq dataset (GSE114007): Articular cartilage samples from patients with OA (red) versus healthy controls (blue) show clear separation along PC1/PC2, indicating distinct transcriptomic profiles. (B) Alzheimer’s disease (AD) microarray dataset (GSE122063): Frontal cortex samples from patients with AD (red) versus controls (blue) exhibit partial separation along PC1, reflecting both shared and heterogeneous transcriptional changes.

PCA analysis showed a clear separation of diseased and control samples in OA, while in AD, a similar grouping trend was observable, albeit less distinct (Fig. 2A and B). OA articular cartilage tissue samples formed distinct clusters relative to healthy controls along the first 2 PCs, indicating major transcriptomic shifts. In AD frontal cortex tissues, sample clusters revealed a partial separation from controls, indicating both shared and heterogeneous transcriptional remodeling within this brain region.

Differential expression analysis identified extensive transcriptional changes in both pathological states. Specifically, OA cartilage exhibited 765 up-regulated and 645 down-regulated genes relative to controls, while AD cortex showed 236 up-regulated and 482 down-regulated genes. Comparative cross-disease analysis revealed 60 overlapping DEGs between datasets, suggesting common molecular features underlying these age-associated diseases (Data File S1-S1).

Functional enrichment of the 60 overlapping DEGs identified stress response, ECM remodeling, and immune regulation terms among shared DEGs. Gene Ontology (GO) biological process analysis showed enrichment in transcriptional regulation in response to stress, synapse pruning, cell junction disassembly, and regulation of macrophage and myeloid cell differentiation. GO cellular component analysis identified ECM structures (collagen-containing matrix and blood microparticles) and neuronal compartments (axon, perikaryon, and growth cone). GO molecular function analysis identified receptor for advanced glycation end-products (RAGE) binding, signaling receptor activator activity, calcium-dependent protein binding, and ECM structural constituents. Kyoto Encyclopedia of Genes and Genomes (KEGG) pathway analysis showed enrichment in prion disease pathways, efferocytosis, glycolysis/gluconeogenesis, peroxisome-proliferator-activated receptor (PPAR) signaling, and complement/coagulation cascades (Data File S1-S2 and Fig. S3).

PPI network analysis identified key hub genes including S100 calcium-binding protein A4 (*S100A4*), gamma-enolase (*ENO2*), and early growth response 1 (*EGR1*). Notably, *S100A4* was consistently up-regulated in both OA and AD, whereas *ENO2* and *EGR1* were down-regulated. Additional markers such as heat shock protein family A (*Hsp70*) member 1A (*HSPA1A*) and Sp1 transcription factor (*SP1*) were particularly enriched within specific chondrocyte subpopulations, underscoring cellular heterogeneity in pathway regulation (Data File S1-S2 and Fig. S4).

### Identification of SUGS

Within the universe of jointly detected genes, we identified 18 genes that were significantly up-regulated in both the AD and OA datasets. A one-sided hypergeometric test indicated that this overlap was greater than expected by chance (*P* = 1.78 × 10^−4^), supporting nonrandom concordance in the up-regulated subset. In contrast, the overlap of down-regulated genes was not significant (*P* = 0.66). Across all jointly detected genes, cross-dataset effect-size concordance was weak: Spearman’s rank correlation *ρ* = −0.0387 (*P* = 5.65 × 10^−5^), and the proportion of directionally concordant genes was 0.477 (near the random expectation of 0.5). This indicates low global concordance typical of cross-platform comparisons, while the shared up-regulated subset nonetheless shows clear and biologically meaningful agreement.

Thus, given the statistically significant overlap, we next focused on these 18 shared up-regulated genes, hereafter referred to as the SUGS. Concentrating on this shared gene set reduces interpretative complexity and allows downstream analyses to target transcriptional programs shared across OA cartilage and AD cortex. Because these datasets were generated from independent cohorts and distinct tissues, the SUGS should be interpreted as evidence of cross-disease molecular convergence rather than direct evidence of intertissue communication, temporal sequence, or causal propagation.

### Single-cell transcriptomic analysis of OA cartilage highlights Fibrochondrocyte_1 as a putative sender-like hub within inferred intratissue networks

**Fig. 3.**
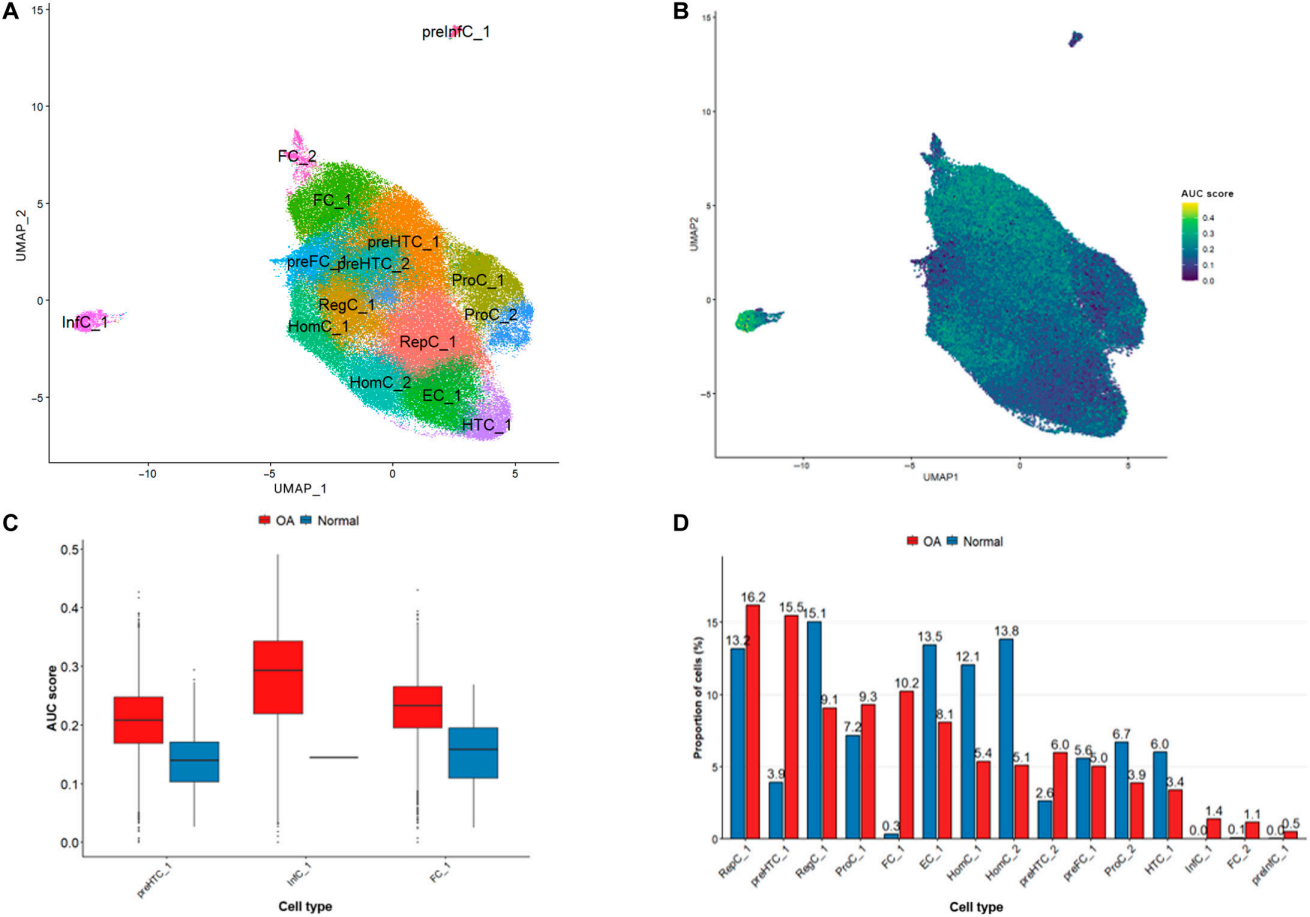
Identification of shared up-regulated gene set (SUGS) activity in osteoarthritis (OA) cartilage. (A) Uniform Manifold Approximation and Projection (UMAP) visualization of single-cell RNA sequencing (scRNA-seq) data (GSE255460) showing distinct chondrocyte and stromal subpopulations in human knee articular cartilage. (B) SUGS enrichment was predominantly observed in prehypertrophic chondrocytes (preHTC_1) and fibrochondrocytes (FC_1), with inflammatory chondrocyte subtypes (InfC_1 and preInfC_1) appearing specifically in OA samples. (C) AUCell-based scoring of SUGS activity across individual OA (red) and normal (blue) cells. (D) Relative cell type proportions in OA and normal cartilage, showing expansion of FC_1, InfC_1, preInfC_1, ProC_1, and preHTC_1, with depletion of homeostatic chondrocytes.

Single-cell transcriptomic data from OA articular cartilage underwent rigorous preprocessing and quality controlled for downstream analysis. Dimensionality reduction via Uniform Manifold Approximation and Projection (UMAP) and subsequent clustering identified distinct cellular populations across OA and control samples (Fig. 3A). Analysis of SUGS pathway activity at the single-cell level revealed disease-specific alterations (Fig. 3B).

Notably, inflammatory chondrocyte subtypes (InfC_1), fibrochondrocytes (FC_1), and prehypertrophic chondrocytes (preHTC_1) exhibited markedly elevated SUGS activity. Group comparison further revealed that their SUGS activity was consistently higher in the OA group than in the normal group (Fig. 3C and Fig. S5).

Comparative analysis of cell type proportions between OA tissues and healthy controls demonstrated an expansion of fibrochondrocytes (FC_1 and FC_2), reparative chondrocytes (RepC_1), inflammatory chondrocyte subtypes (InfC_1 and preInfC_1), ProC (proliferation chondrocytes), and prehypertrophic chondrocytes (preHTC_1 and preHTC_2) in OA, accompanied by a substantial depletion of homeostatic chondrocytes (HomC_1 and HomC_2). Donor-aware differential abundance testing indicated that although FDR values did not reach statistical significance (likely due to limited sample size), FC_1, HomC_2, and HomC_1 showed statistically significant differences at the nominal *P* < 0.05 threshold. Moreover, the nominal *P* values of InfC_1 (*P* = 0.052) and FC_2 (*P* = 0.062) were also close to the significance threshold (Fig. 3D and Data File S1-S3).

**Fig. 4.**
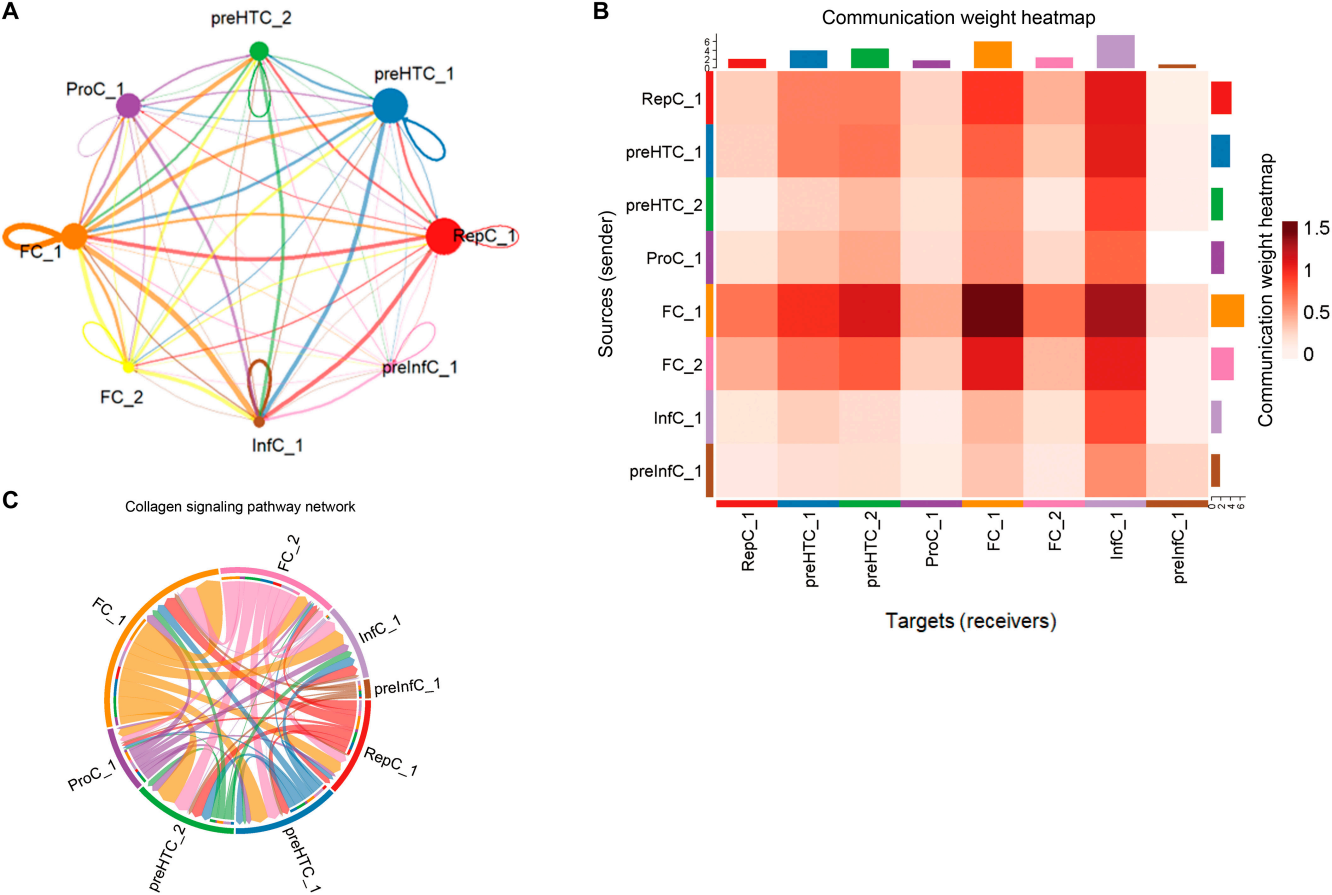
Inferred ligand–receptor network analysis in osteoarthritis (OA) cartilage. (A) Network diagram of inferred intratissue intercellular communication, identifying FC_1 as the most prominent sender-like population within the OA dataset. (B) Heatmap of ligand–receptor pair interactions, showing strong predicted outgoing signals from FC_1 to InfC_1, ProC_1, and preHTC_1. (C) Pathway-level analysis highlighting collagen signaling as the principal predicted outgoing pathway from FC_1 within OA cartilage.

To capture a comprehensive disease-associated cell–cell communication landscape, we incorporated only OA-derived cells belonging to the 8 subpopulations (FC_1, FC_2, RepC_1, InfC_1, preInfC_1, ProC, preHTC_1, and preHTC_2) into the downstream analysis. Ligand–receptor interaction mapping identified the FC_1 fibrochondrocyte subpopulation as the dominant sender, while InfC_1 emerged as the principal-signal-receiving subpopulation (Fig. 4A and B). To further quantify these observations, we additionally computed quantitative centrality metrics for sender–receiver strength, which consistently confirmed the same conclusion (Data File S1-S4).

Pathway analysis showed collagen signaling as the most enriched outgoing pathway from FC_1 (Fig. 4C). CellChat analysis identified fibrochondrocytes, particularly FC_1, as a central component of the inferred SUGS-driven intercellular communication network in OA. Interestingly, FC_1, preHTC_1, and InfC_1 not only displayed increased SUGS activity in OA but also demonstrated an expansion in cellular abundance (with InfC_1 being an OA-specific subtype completely absent in normal cartilage).

To further elucidate the functional identity of the FC_1, preHTC_1, and InfC_1, we examined its top DEGs. Notably, collagen type I alpha 1 (*COL1A1*) exhibited a high average log_2_FC (4.49-fold), with expression detected in 87.1% of FC_1 cells compared to 13.7% in other clusters. Similarly, matrix metalloproteinase-2 (*MMP2*) showed strong up-regulation (log_2_FC = 4.20; 80.6% versus 12.6%), while COL14A1 was also prominently expressed (log_2_FC = 3.91; 72.2% versus 12.8%). These patterns indicate enrichment of ECM components and fibrosis-associated transcripts within FC_1, consistent with fibrotic remodeling in OA cartilage. For the InfC_1 subpopulation, key innate immune and antigen presentation genes, including human leukocyte antigen class II histocompatibility antigen, DQ alpha 1 chain (*HLA-DQA1*), cytochrome B-245 beta chain (*CYBB*), and macrophage scavenger receptor 1 (*MSR1*), showed extremely high average log_2_FC values and were detected in a large fraction of InfC_1 cells. For the preHTC_1 subpopulation, several cartilage- and matrix-associated genes were prominently enriched. ABI family member 3 binding protein (*ABI3BP*) and cartilage acidic protein 1 (*CRTAC1*) showed strong up-regulation and high detection frequency, together with matrix-related genes such as collagen type XV alpha 1 (*COL15A1*) and a metallopeptidase with thrombospondin type 1 motif 6 (*ADAMTS6*) (Data File S1-S5).

Pseudobulk analysis further revealed distinct transcriptional differences between OA and healthy samples within the FC_1 and preHTC_1 subpopulations. In FC_1 cells, OA samples showed down-regulation of interferon-induced protein with tetratricopeptide repeats 2 (*IFIT2*), interferon-induced protein with tetratricopeptide repeats 3 (*IFIT3*), and mitochondrial ribosomal RNA genes *MT-RNR1* (mitochondrially encoded 12*S* rRNA) and *MT-RNR2* (mitochondrially encoded 16*S* rRNA), along with changes in cytoskeletal-related genes such as septin family (*SEPTIN2*, *SEPTIN7*, and *SEPTIN11*) and myocilin (*MYOC*). In contrast, preHTC_1 cells exhibited up-regulation of cartilage- and matrix-associated genes, including tenascin (*TNC*), and high-temperature requirement (*HtrA*) serine peptidase 1 (*HTRA1*). In addition, the bone morphogenetic protein (BMP) antagonist chordin-like 2 (*CHRDL2*) was significantly down-regulated, potentially alleviating the inhibition of BMP signaling in this subset. Notably, several genes displayed opposing regulation between subpopulations, highlighting cell-type-specific transcriptional remodeling. These findings suggest divergent molecular responses across chondrocyte subtypes in OA (Data File S1-S6).

Since FC_1 and InfC_1 were identified as key subpopulations and also acted as major senders/receivers in the cell–cell communication network, we further examined the overlap between their SUGS genes and the corresponding ligand–receptor signaling pathways. However, no SUGS-related genes were detected within these communication signals.

### Single-cell transcriptomic analysis of AD cortex highlights Oligodendrocyte_3 as a putative receiver-like hub within inferred intratissue networks

**Fig. 5.**
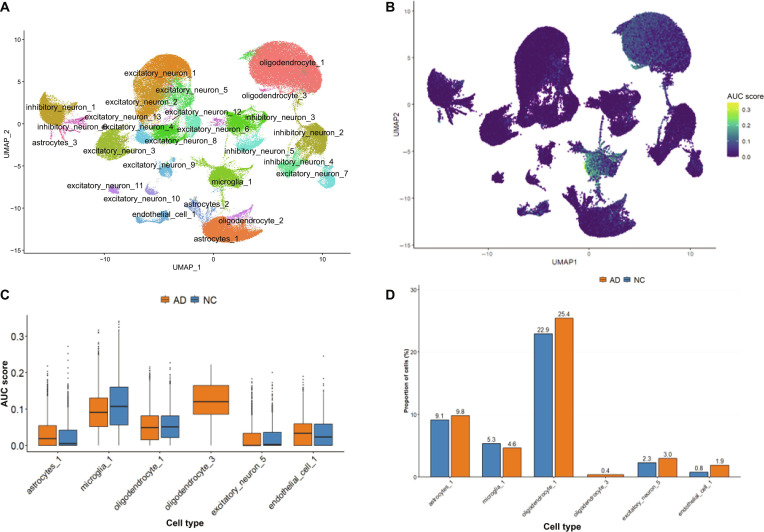
Single-cell analysis of shared up-regulated gene set (SUGS) activity in Alzheimer’s disease (AD) cortex. (A) Uniform Manifold Approximation and Projection (UMAP) visualization of single-cell RNA sequencing (scRNA-seq) data (GSE157827) showing distinct cellular subpopulations in the AD cortex. (B) SUGS enrichment was primarily observed in oligodendrocyte_1, oligodendrocyte_3, excitatory_neuron_5, astrocytes_1, microglia_1, and endothelial_cell_1 clusters, with oligodendrocyte_3 detected exclusively in the AD cortex. (C) Box plots of SUGS activity scores across cell types, showing higher scores in oligodendrocyte_3, astrocytes_1, excitatory_neuron_5, and endothelial_cell_1 in AD than in controls. (D) Proportion analysis of AD versus control samples, showing increased representation of astrocytes_1, excitatory_neuron_5, and endothelial_cell_1, with oligodendrocyte_3 detected exclusively in AD.

UMAP visualization of scRNA-seq data from the AD prefrontal cortex revealed that SUGS activity was concentrated within specific glial and neuronal subpopulations (Fig. 5A and B). The microglia_1 and oligodendrocyte_3 subpopulations exhibited higher SUGS activity. In addition, based on a combined assessment of area under the curve (AUC) scores and cell abundances, the subpopulations oligodendrocyte_1, astrocytes_1, excitatory_neuron_5, and endothelial_cell_1 were selected for comparative analyses between AD samples and healthy controls (Fig. 5C and Fig. S6). Of particular note, the oligodendrocyte_3 subpopulation showed pronounced up-regulation exclusively in AD, while astrocytes_1 maintained comparable proportions between AD and controls but displayed markedly increased SUGS transcriptional activity, indicative of transcriptional dysregulation without changes in cell abundance.

Subsequently, complementary cell proportion analysis focusing on the 6 selected subpopulations showed that, in terms of overall abundance, 5 of the 6 subpopulations were expanded in AD, with the exception of microglia_1. Notably, oligodendrocyte_3 was detected exclusively in the disease group, representing an AD-specific subpopulation (Fig. 5D). However, donor-aware differential abundance testing revealed that none of these changes reached statistical significance (*P* > 0.05) (Data File S1-S3). This may be attributable to the limited sample size or the intrinsic complexity of cellular composition in brain tissue.

**Fig. 6.**
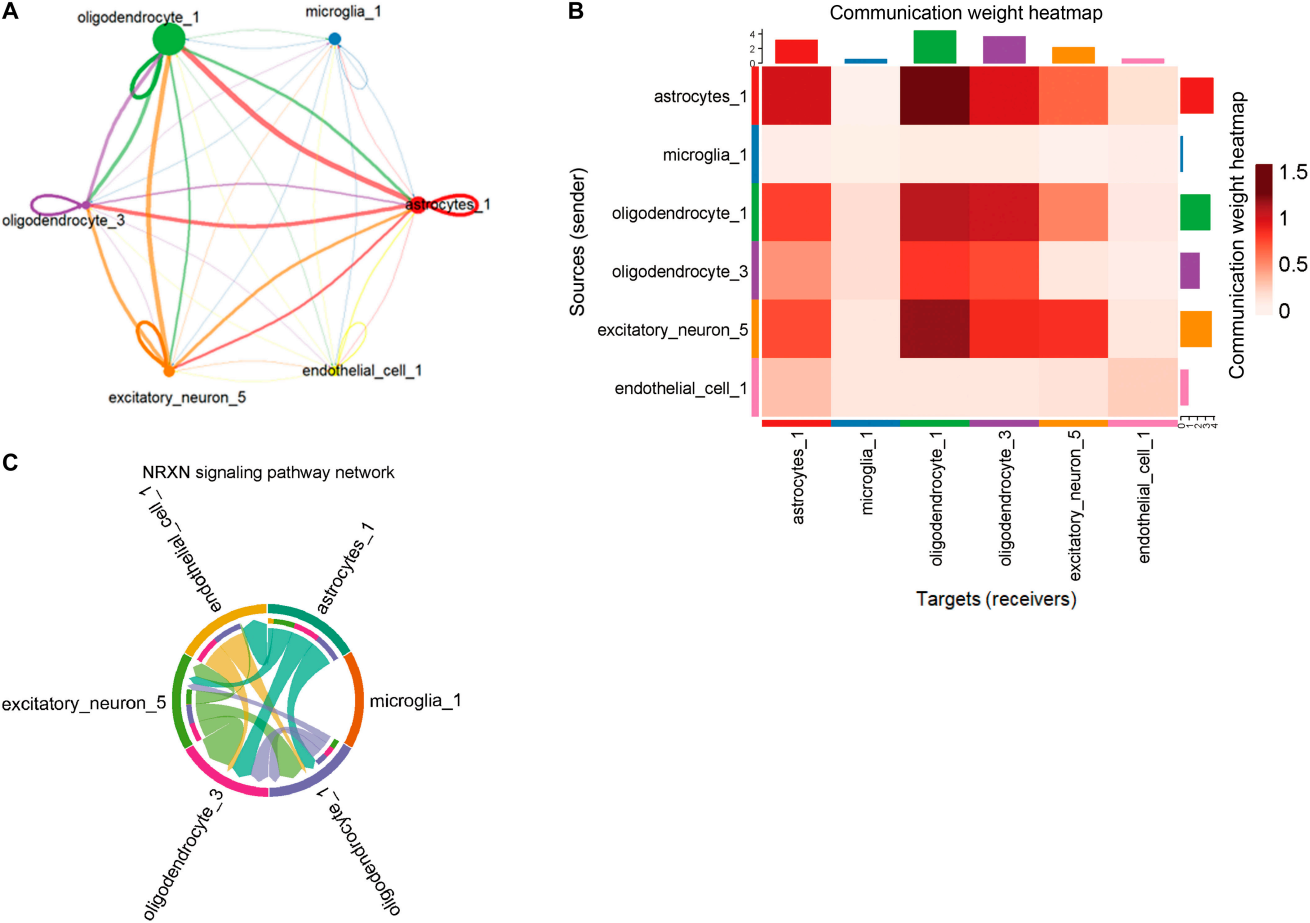
Inferred cell–cell communication networks in Alzheimer’s disease (AD) cortex. (A) Network diagram of inferred ligand–receptor interactions, highlighting oligodendrocyte_3 as a prominent-receiver-like population within the AD dataset. (B) Heatmap visualization of inferred signaling strength, identifying oligodendrocyte_3 as a major predicted target of intercellular communication. (C) Pathway-level analysis showing neurexin (NRXN) signaling as the most enriched inferred pathway, with excitatory_neuron_5 as the dominant-sender-like population and oligodendrocyte_3 as a prominent-receiver-like population.

Ligand–receptor interaction mapping identified the astrocytes_1 subpopulation as the dominant sender, whereas oligodendrocyte subtypes (oligodendrocyte_1 and oligodendrocyte_3) emerged as the principal-signal-receiving populations (Fig. 6A and B). Notably, oligodendrocyte_3, which is one of our key subpopulations of interest, was highlighted by the quantitative centrality metrics as a major signal receiver, ranking second only to oligodendrocyte_1 (Data File S1-S4).

Pathway-level analysis highlighted significant enrichment of the neurexin (*NRXN*) signaling pathway (Fig. 6C). Within this pathway, excitatory_neuron_5 emerged as the dominant sender population, transmitting NRXN-related ligands to multiple subtypes, with oligodendrocyte_3 serving as the principal receiver. Astrocytes and endothelial cells also contributed to the NRXN communication circuitry.

Differential expression analysis also showed that oligodendrocyte_3 is enriched for heat shock and molecular chaperone genes, most prominently Hsp70 member 1B (*HSPA1B*) (avg_log_2_FC = 4.35; 75.8% versus 13.4%), *HSPA1A* (avg_log_2_FC = 4.24; 86.9% versus 20.6%) and crystallin, alpha B (*CRYAB*) (avg_log_2_FC = 3.77; 78.5% versus 24.0%), indicating a stressed, proteostasis-impaired oligodendrocyte state in AD cortex (Data File S1-S5).

Pseudobulk analysis further revealed transcriptional differences between AD and healthy samples within these key subpopulations (Data File S1-S6). In astrocytes_1, AD-associated nominal changes were observed for genes involved in inflammation, lipid metabolism, and vascular signaling. Notably, apolipoprotein E (*APOE*) and vascular endothelial growth factor A (*VEGFA*) were up-regulated, consistent with their roles in AD risk, neuroinflammation, and BBB disruption. Conversely, the anti-inflammatory transcription factor Krüppel-like factor 2 (*KLF2*) was down-regulated. In oligodendrocyte_1, altered expression of catenin alpha 2 (*CTNNA2*) and purinergic receptor P2Y1 (*P2RY1*) suggests possible dysregulation of neuron–glia communication pathways relevant to synaptic integrity and inflammatory modulation.

We also examined the potential roles of SUGS genes within the communication networks of the 6 subpopulations. However, similarly, no SUGS-related genes were detected within these ligand–receptor signaling interactions. This suggests that SUGS genes may not primarily function through cell–cell communication mechanisms, or, alternatively, this absence may be related to the background constraints of the CellChat ligand–receptor database.

### Neurovascular- and endothelial-associated pathway activity in OA and AD

Although the current data do not provide direct evidence for cross-tissue molecular transmission between OA and AD, we nevertheless examined the single-cell-level activity of several BBB-associated KEGG pathways, including tight junction, adherens junction, leukocyte transendothelial migration, VEGF signaling pathway, Wnt signaling pathway, ECM–receptor interaction, and focal adhesion.

In OA, ECM–receptor interaction and focal adhesion showed higher pathway activity scores in the key disease-relevant subpopulations preHTC_1 and FC_1, while InfC_1 displayed elevated scores for pathway categories related to tight junction, adherens junction, and leukocyte transendothelial migration. Several of these pathway scores were higher in OA than in normal samples within the selected subpopulations.

In AD, astrocytes_1 and endothelial_cell_1 also showed signal across selected neurovascular- and endothelial-associated pathway categories, including adherens junction, VEGF signaling, and Wnt signaling. Marker analysis of endothelial_cell_1 supported its endothelial identity, with top markers including Claudin-5, Fms-related tyrosine kinase-1 (*FLT1*), and Sex-determining region Y box 18 (*SOX18*). Exploratory pseudobulk analysis further identified genes of potential relevance to neurovascular and adhesion-related biology within astrocytes_1 and endothelial_cell_1; however, these findings were not sufficient to establish a robust BBB dysfunction or endothelial activation signature. Accordingly, these results should be interpreted as limited transcriptomic support for neurovascular/endothelial-associated molecular context within the AD dataset, rather than as evidence of altered BBB permeability, endothelial-mediated OA-to-brain signaling, or direct cross-tissue communication. Instead, they provide a cautious, hypothesis-generating framework in which shared OA–AD transcriptional programs may intersect with ECM-, adhesion-, and neurovascular-associated pathways.

The detailed AUCell-based neurovascular/endothelial pathway scores and box plots are provided in Figs. S7 to S10.

## Discussion

This study presents an integrative cross-disease transcriptomic analysis combining bulk and single-cell datasets from OA and AD to identify aging-associated cellular programs shared between these prevalent disorders. At the bulk transcriptomic level, OA and AD samples exhibited distinct transcriptional profiles compared with controls, and 60 overlapping DEGs were identified, suggesting partial convergence of disease-associated molecular alterations, including chronic inflammation, immune modulation, metabolic dysregulation, and ECM remodeling. However, these shared cellular hubs should be viewed as biologically relevant features of cross-disease transcriptomic convergence rather than as validated biomarkers or immediately therapeutically actionable nodes, and their translational relevance remains to be established in independent cohorts and functional studies.

Among the 60 overlapping DEGs, 18 genes were identified as being significantly up-regulated in both OA and AD datasets and constituting the shared up-regulated gene set (SUGS) that served as the basis for our downstream functional analyses. Functional enrichment of the 18 SUGS genes indicated convergence on structural remodeling, vascular-associated signaling, metabolic adaptation, and impaired clearance processes. ECM-related components, including collagen-associated structures and microparticles, coenriched alongside neuronal cellular compartments such as axons and growth cones, consistent with tissue remodeling responses to chronic stress in both joint and brain environments. In OA, cartilage ECM degradation generates bioactive fragments and damage-associated molecular patterns that sustain inflammatory signaling [4]. In AD, synaptic degeneration and perineuronal net disruption contribute to neuronal vulnerability [10,35]. Together, these observations align with the conceptual “brain–joint axis” model proposed by Rabie et al. [2], which describes potential neuroimmune and systemic interactions linking peripheral and central pathology, although it remains unclear whether these shared features reflect direct communication or parallel manifestations of aging-associated inflammatory stress.

Enrichment of receptor–ligand interaction terms, including pathways involving RAGE, highlights vascular and inflammatory signaling components implicated in both OA and AD. Experimental studies have demonstrated that advanced glycation end-products and S100 proteins can engage endothelial RAGE receptors and influence neuroinflammatory responses under certain conditions [6,36]. In addition, systemic inflammation has been associated with altered BBB integrity in aging and neurodegenerative contexts [2,37]. In the present analysis, vascular-associated GO terms were identified in the shared DEG set and endothelial_cell_1 was among the AD-relevant subpopulations, but these observations remain indirect.

To further assess potential neurovascular context, we performed a targeted transcriptomic interrogation of selected neurovascular- and endothelial-associated KEGG pathways across key OA and AD subpopulations. These analyses provided limited pathway-level support for neurovascular/endothelial-associated molecular context and did not establish BBB dysfunction, endothelial activation, or endothelial-specific cross-tissue signaling. Accordingly, our data support only a cautious interpretation that neurovascular processes may represent one plausible component of the shared OA–AD transcriptional landscape.

Metabolic stress and impaired clearance pathways also emerged as overlapping features. Both OA chondrocytes and AD neurons have been reported to undergo glycolytic metabolic reprogramming under chronic stress [38,39]. Dysregulation of PPAR signaling has been implicated in cartilage degeneration [40,41], as well as metabolic dysfunction in neurodegeneration. Furthermore, compromised efferocytosis in synovial macrophages [42] and microglia [43] has been associated with sustained inflammatory environments in both diseases. These convergent pathways may reflect broader aging-associated alterations in immune regulation and metabolic homeostasis rather than direct inter-organ signaling.

Single-cell analyses further revealed cell-type-specific transcriptional programs within each tissue. In OA cartilage, fibrochondrocytes (particularly the FC_1 subset) displayed enrichment of ECM remodeling genes and occupied central positions within inferred intratissue communication networks [26,44]. The InfC_1 subset was characterized by markedly increased expression of key innate immune and antigen presentation genes such as *HLA-DQA1*, *CYBB*, and *MSR1*, with high detection rates across the cell population, supporting its interpretation as a proinflammatory, antigen-presenting, macrophage-like chondrocyte subset with prominent innate immune activity, pathogen response features, and chemokine-driven recruitment capacity. Within the OA cartilage microenvironment, this population is likely to contribute to the amplification of local inflammation and to the dynamic interplay between immune activation and ECM remodeling. In contrast, the preHTC_1 subpopulation was characterized by increased expression of *TNC* and *HTRA1*, consistent with active matrix remodeling, alongside significant down-regulation of *CHRDL2*, a known antagonist of BMP signaling, suggesting reduced inhibitory control over this pathway and a complex OA-associated cell state in which repair-related and degradative signals coexist.

In the AD cortex, oligodendrocyte_3 exhibited elevated expression of stress-response and proteostasis-related genes, consistent with emerging evidence of oligodendrocyte vulnerability in AD [45]. Within the AD dataset, NRXN-associated signaling was identified as a prominent intratissue communication pathway, in agreement with its established role in synaptic adhesion and neuronal–glial interaction [46,47].

Prior literature has implicated ECM–β1 integrin and fibronectin 1–Toll-like receptor 4 (TLR4) signaling in inflammatory amplification and vascular regulation [48–52]. The present data are compatible with this broader literature insofar as ECM-, integrin-, and TLR-associated transcripts recur across the bulk and single-cell analyses, but the datasets do not include circulating ligand measurements, BBB permeability readouts, or direct endothelial signaling assays. The ECM–integrin–TLR axis should therefore be regarded as a biologically plausible interpretive framework rather than a demonstrated mechanism linking OA and AD, providing mechanistic context and testable hypotheses rather than causal proof.

### A hypothesis-generating systems framework for OA–AD comorbidity

Our findings support a cell-resolved, hypothesis-generating framework in which distinct cellular populations in OA cartilage and AD cortex are enriched for partially overlapping stress- and inflammation-associated transcriptional programs. Because the analyses rely on cross-sectional transcriptomic datasets from independent cohorts and distinct tissues, this framework represents a conceptual model of OA–AD comorbidity, not proof of inter-organ communication. In OA cartilage, the FC_1 subset was enriched for ECM remodeling transcripts, including *COL1A1*/*COL1A2*, *COL14A1*, and *MMP2*, consistent with cartilage dedifferentiation and matrix remodeling previously described in OA [44,53]. In related contexts, ECM degradation products and damage-associated molecular patterns have been shown to contribute to inflammatory signaling beyond the local tissue environment [4].

In the AD cortex, oligodendrocyte_3 displayed elevated expression of stress-related and chaperone genes, including *HSPA1B*, *HSPA1A*, and *CRYAB*, in line with proteostatic and metabolic stress programs described in neurodegenerative disease [27,54]. Independent ligand–receptor inference within the AD dataset further identified this subpopulation as a prominent receiver within neuronal–glial communication networks, particularly in relation to NRXN-associated signaling, which may reflect broader synaptic and metabolic vulnerability in the AD cortex rather than a strictly oligodendrocyte-specific mechanism [46,47]. Endothelial-associated transcriptional context was also observed in the AD dataset but remained exploratory and inferential. Taken together, these intratissue findings support a systems-level model in which matrix remodeling, chronic inflammation, and altered intercellular communication converge on partially shared molecular programs across OA cartilage and AD cortex, without demonstrating direct inter-organ signaling. In this context, sender–receiver relationships refer to relative centrality within inferred intratissue communication networks rather than experimentally validated directional signaling, and the inferred pathways should be regarded as biologically relevant candidates requiring orthogonal validation and further perturbational study.

### Limitations and future directions

While our study presents a refined, directional sender–receiver model that enhances the understanding of OA–AD cross-talk at a cell-type-specific level, several limitations should be acknowledged. First, the cross-disease transcriptomic overlap identified here should not be interpreted as the sole explanation for OA–AD comorbidity. Rather, the present data support ECM remodeling and inflammatory stress as one plausible component of a broader multifactorial process, as suggested by convergence of the SUGS, the FC_1 program in OA, and the stress-associated oligodendrocyte_3 state in AD on ECM remodeling, inflammatory signaling, and cellular stress, while alternative and potentially interacting mechanisms remain plausible. The enrichment of vascular-associated terms in the shared DEG analysis, together with RAGE-related signaling and the inclusion of endothelial_cell_1 among the AD-relevant subpopulations, is compatible with neurovascular or BBB-related mechanisms, but the available datasets do not include direct measures of vascular integrity, cerebral perfusion, or BBB permeability and are therefore insufficient to test this possibility directly [55,56]. Likewise, the identified glycolysis/gluconeogenesis and PPAR-related signals are compatible with broader systemic metabolic dysregulation, yet in the absence of subject-level information on obesity, insulin resistance, diabetes, or lipid status, it cannot be determined whether these transcriptional changes reflect shared systemic metabolic drivers or tissue-intrinsic stress responses [57–59].

Shared genetic susceptibility is another plausible contributor, but the present cross-sectional transcriptomic design cannot distinguish inherited risk architecture from downstream disease-state transcriptional convergence, and this question remains unresolved given mixed genetic evidence, including null Mendelian randomization findings alongside reports suggesting that OA may interact with APOE-related AD vulnerability [23,60,61]. Medication-related effects must also be considered, because the analyzed cohorts were independent and medication exposure was not harmonized; long-term nonsteroidal anti-inflammatory drug (*NSAID*) use or other treatments may therefore have influenced the observed disease associations or transcriptomic states, particularly in light of the complex relationship between NSAID exposure and dementia risk reported in the literature [62,63]. Overall, the findings are most consistent with a multifactorial model in which ECM-driven inflammation represents one data-compatible component that may interact with vascular, metabolic, genetic, and treatment-related factors.

Second, the integrative analysis combines cross-sectional bulk RNA-seq, microarray, and scRNA-seq datasets generated from different platforms, tissues, populations, and independent cohorts. Although normalization and batch-correction approaches were applied, cross-platform variability and cohort heterogeneity may still have influenced differential expression results and gene overlap identification. Future studies incorporating matched multitissue sampling from the same individuals would substantially improve inference regarding coordinated systemic responses and any putative cross-tissue relationships.

Third, all intercellular communication inferences were derived from transcriptomic data. CellChat infers potential ligand–receptor communication from gene expression patterns and curated interaction databases, but it does not demonstrate that inferred sender and receiver populations are in physical proximity or functionally interact in situ [34]. In addition, transcript abundance does not necessarily predict protein abundance, as protein levels and signaling output are shaped by posttranscriptional regulation, translation efficiency, protein turnover, and other regulatory processes [64,65]. This limitation is especially relevant for secreted ligands and membrane receptors, whose functional activity depends on multiple posttranscriptional and posttranslational processes, including protein processing, trafficking, localization, and turnover [66–68]. Single-cell dissociation also disrupts native tissue architecture, precluding direct confirmation that the cell populations highlighted in OA cartilage and AD cortex occupy compatible anatomical niches. Accordingly, the sender–receiver relationships and signaling pathways described here should be interpreted as hypothesis-generating transcriptomic predictions rather than confirmed functional communication events. Spatial transcriptomics, multiplexed imaging, orthogonal protein-level validation, and functional studies will be required to determine whether these inferred cellular pairs are anatomically adjacent and engage in true in situ communication.

Furthermore, our analyses were performed independently within each tissue and did not include direct measurements of circulating mediators, BBB integrity, or systemic inflammatory signaling. Consequently, it cannot be concluded that ECM-derived mechanical or stress-related signals themselves cross the BBB, particularly given the highly selective nature of the BBB and the importance of local ECM–integrin interactions for barrier integrity [14,48]. Although BBB dysfunction has been described in aging and AD [37,69], any peripheral effects would more plausibly involve indirect inflammatory or neurovascular mediators rather than direct transfer of ECM-derived mechanical information [4,50]. While endothelial_cell_1 and selected neurovascular/endothelial-associated pathway signals were identified in the AD dataset, these findings remained exploratory and were insufficient to define a robust BBB dysfunction or endothelial activation signature. Future studies integrating spatially resolved approaches, matched peripheral and central biospecimens, proteomic validation, and experimental BBB models will be required to clarify whether the shared transcriptional programs identified here reflect neurovascular involvement, coordinated systemic mechanisms, or parallel tissue responses to chronic aging-associated stress.

## Conclusion

In summary, this integrative bulk and single-cell transcriptomic analysis identified cell-type-specific transcriptional programs that are shared between OA cartilage and AD cortex. Within each tissue, distinct cellular populations were enriched for inflammatory and stress-associated gene expression patterns and occupied central positions within inferred intratissue communication networks. Although these findings do not demonstrate direct inter-organ signaling, they support a systems-level conceptual framework in which peripheral matrix remodeling and central glial stress responses may converge on overlapping molecular pathways during aging. As a hypothesis-generating model, this framework provides a structured basis for future experimental studies aimed at clarifying potential mechanisms underlying OA–AD comorbidity and highlights candidate molecular interactions for further validation, while emphasizing shared cellular programs and communication networks rather than immediate therapeutic actionability.

## Ethical Approval

Not applicable.

## Data Availability

All raw and processed datasets utilized in this study are publicly accessible through the Gene Expression Omnibus under the following accession numbers: OA bulk RNA-seq (GSE114007), AD bulk microarray (GSE122063), OA scRNA-seq (GSE255460), and AD scRNA-seq (GSE157827). Processed data matrices and related analysis scripts will be made available from the corresponding author upon reasonable request. The full analysis pipeline and R scripts are available on GitHub (https://github.com/znGer-cel/Sender-and-Receiver-Cellular-Hubs-as-Potential-Therapeutic-Targets-in-OA-AD-Comorbidity).
