# Supplementary material for: A Systems-Level Transcriptomic Framework Identifies Shared Cellular Hubs in Osteoarthritis and Alzheimer’s Disease
Source: Comput Struct Biotechnol J. 2026 May 13;35(1):0085. doi: 10.34133/csbj.0085 (PMC13168759; doi:10.34133/csbj.0085)
Supplement: Supplementary 1 — Data File S1 Figs. S1 to S10 [file csbj.0085.f1.zip › Supplementary File 2.docx]

***Supporting Information***

**Systems-Level Transcriptomic Framework Identifies Shared Cellular Hubs in Osteoarthritis and Alzheimer’s Disease**

^1†^Zhangzheng Wang, ^1†^Krisztián Juhász Zoltán, ^1^Csaba Matta, ^1^Luca Paluska, ^1^ Ahmed Al-Mnaseer, ^1†^Roland Takács, ^1†^László Ducza

**Contents**

| *Figure S1.* | Cell Subpopulation Annotation and Classical Marker Expression for OA |
| --- | --- |
| *Figure S2.* | Cell Subpopulation Annotation and Classical Marker Expression for AD |
| *Figure S3.* | Functional enrichment analysis of shared DEGs |
| *Figure S4.*  *Figure S5.*  *Figure S6.* | Protein–protein interaction (PPI) analysis of shared DEGs  SUGS activity (AUC score) across OA cell subpopulations  SUGS activity (AUC score) across AD cell subpopulations |
| *Figure S7.*  *Figure S8.*  *Figure S9.* | Neurovascular- and Endothelial-Associated Pathway Activity (AUC score) across OA cell subpopulations  Boxplots of Neurovascular- and Endothelial-Associated Pathway Activity in Specific OA Cell Subclusters  Neurovascular- and Endothelial-Associated Pathway Activity (AUC score) across AD cell subpopulations |
| *Figure S10.* | Boxplots of Neurovascular- and Endothelial-Associated Pathway Activity in Specific AD Cell Subclusters |


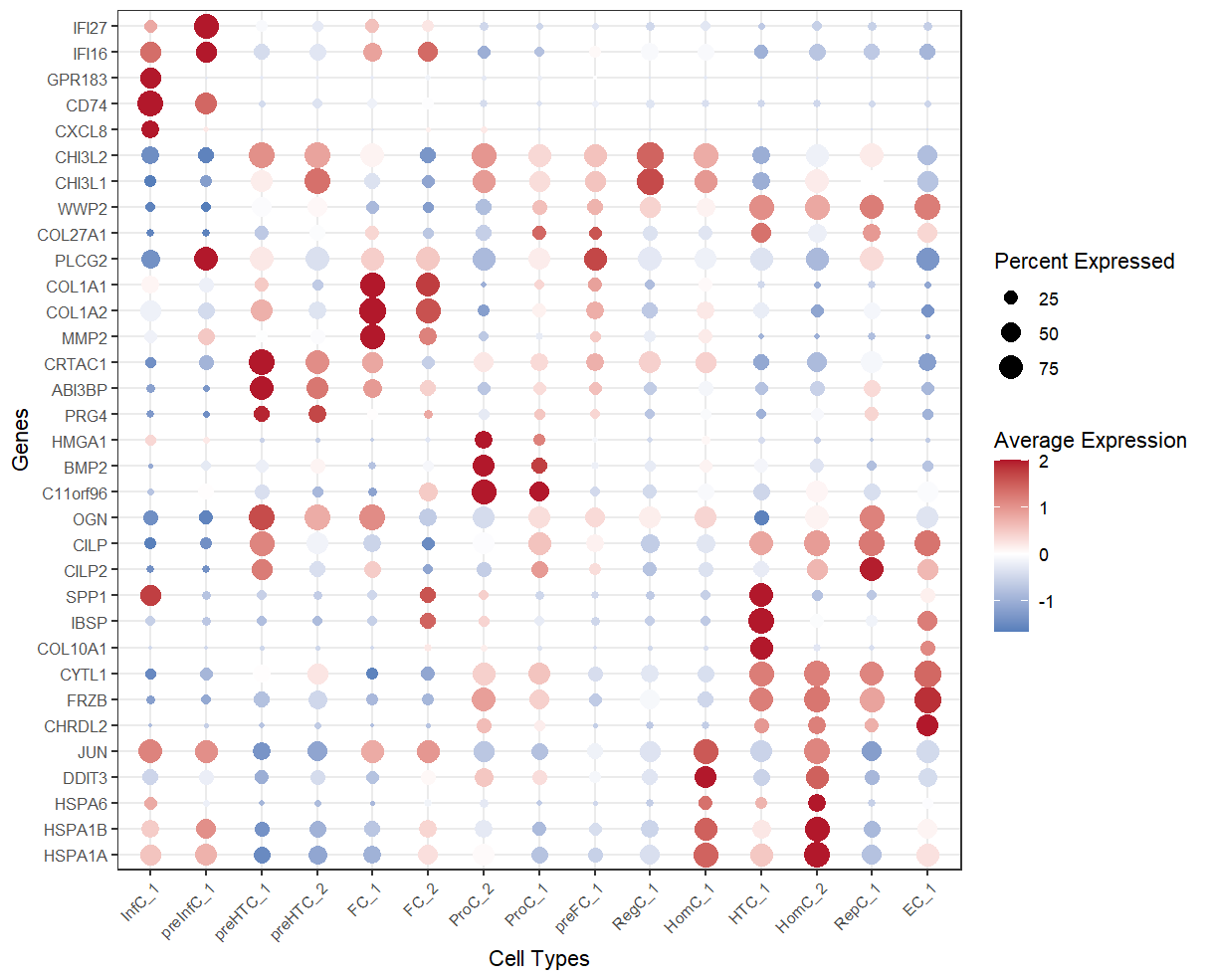


*Figure S1. Cell subpopulation annotation and classical marker expression for OA*

Cell identities were assigned using a marker‑based strategy guided by canonical markers reported in the source studies. Clusters were annotated by concordance with curated marker sets: **HomC (homeostasis chondrocytes)**—*HSPA1A*, *HSPA1B*, *HSPA6*, *DDIT3*, *JUN*; **EC (effector chondrocytes)**—*CHRDL2*, *FRZB*, *CYTL1*; **HTC (hypertrophic/terminal chondrocytes)**—*COL10A1*, *IBSP*, *SPP1*; **RepC (reparative chondrocytes)—***CILP2*, *CILP*, *OGN*; **ProC (proliferation chondrocytes)**—*C11orf96*, *BMP2*, *HMGA1*; preHTC—PRG4, ABI3BP, CRTAC1; **FC (fibrocartilage chondrocytes)**—MMP2, COL1A2, COL1A1; **preFC**—*PLCG2*, *COL27A1*, *WWP2*; **RegC (regulator chondrocytes)**—*CHI3L1*, *CHI3L2*; **InfC (inflammatory chondrocytes)**—*CXCL8*, *CD74*, *GPR183*; **preInfC**—*IFI16*, *IFI27*.


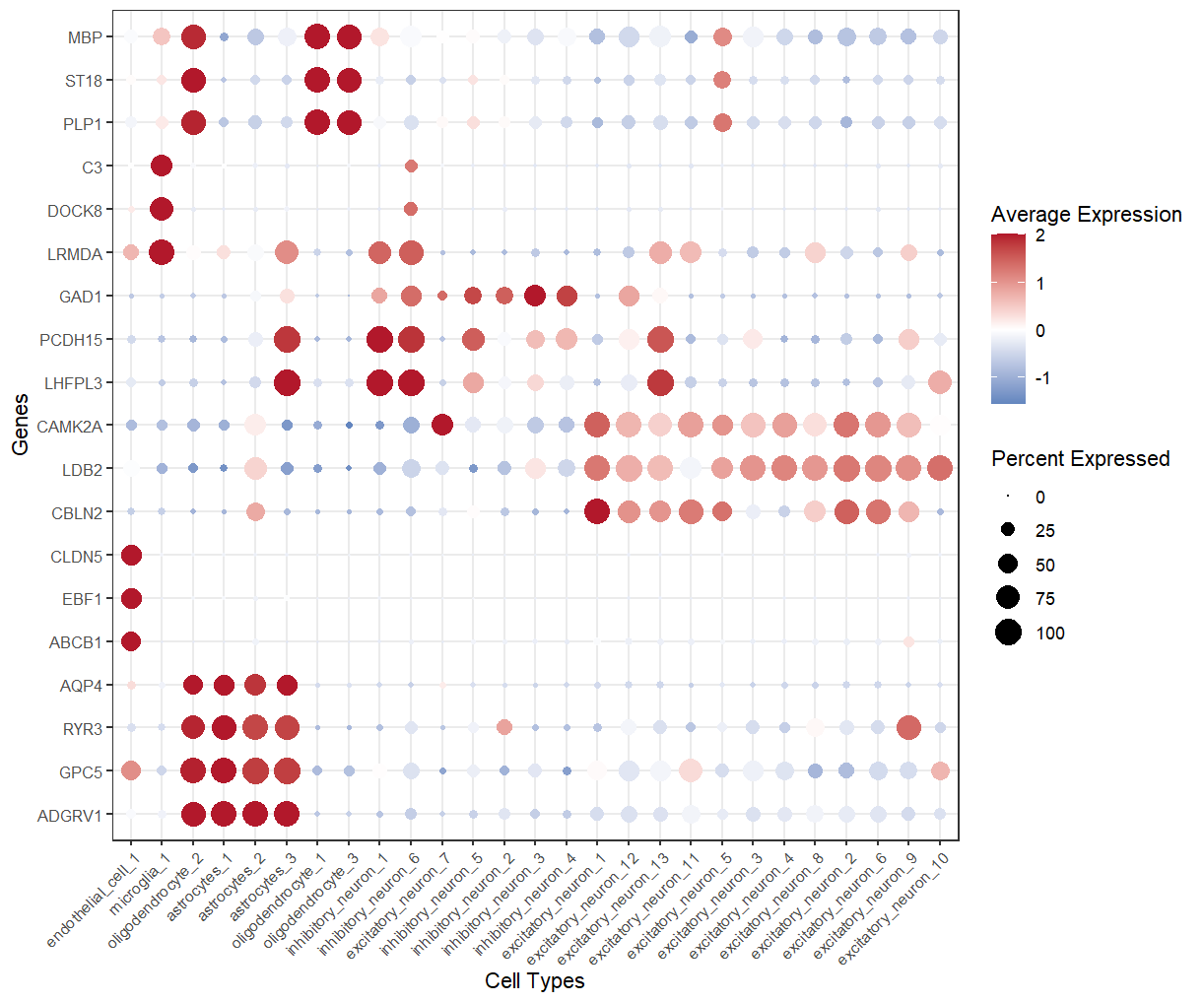


*Figure S2. Cell subpopulation annotation and classical marker expression for AD.*

Cell identities were assigned using a markerbased strategy guided by canonical‑ markers reported in the source studies. Clusters were annotated by concordance with curated marker sets as follows: **astrocytes**—*ADGRV1, GPC5, RYR3, AQP4*; **endothelial cells**—*ABCB1, EBF1, CLDN5*; **excitatory neurons**—*CBLN2, LDB2, CAMK2A*; **inhibitory neurons**—*LHFPL3, PCDH15, GAD1*; **microglia**—*LRMDA, DOCK8, C3*; **oligodendrocytes**—*PLP1, ST18, MBP*. Marker expression was visualized with dot plot; ambiguous clusters were manually reviewed using complementary lineage markers to ensure consistent labeling across samples.


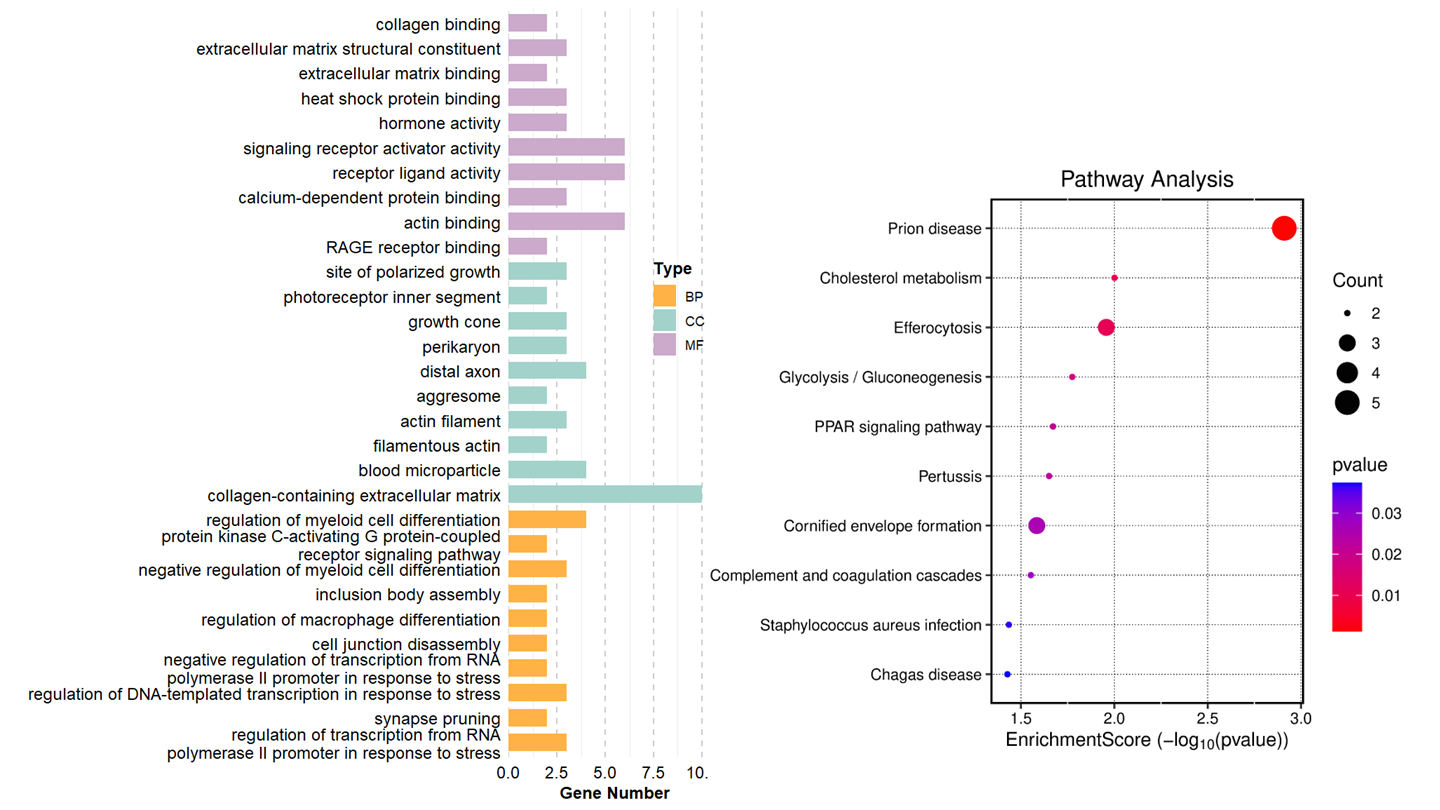


*Figure S3. Functional enrichment analysis of shared DEGs.*

(A) Overview of significantly enriched biological processes, molecular functions, and cellular components identified by Gene Ontology (GO) analysis.

(B) KEGG pathway enrichment analysis showing notably enriched pathways, including prion disease, efferocytosis, glycolysis/gluconeogenesis, PPAR signaling, and complement/coagulation cascades.


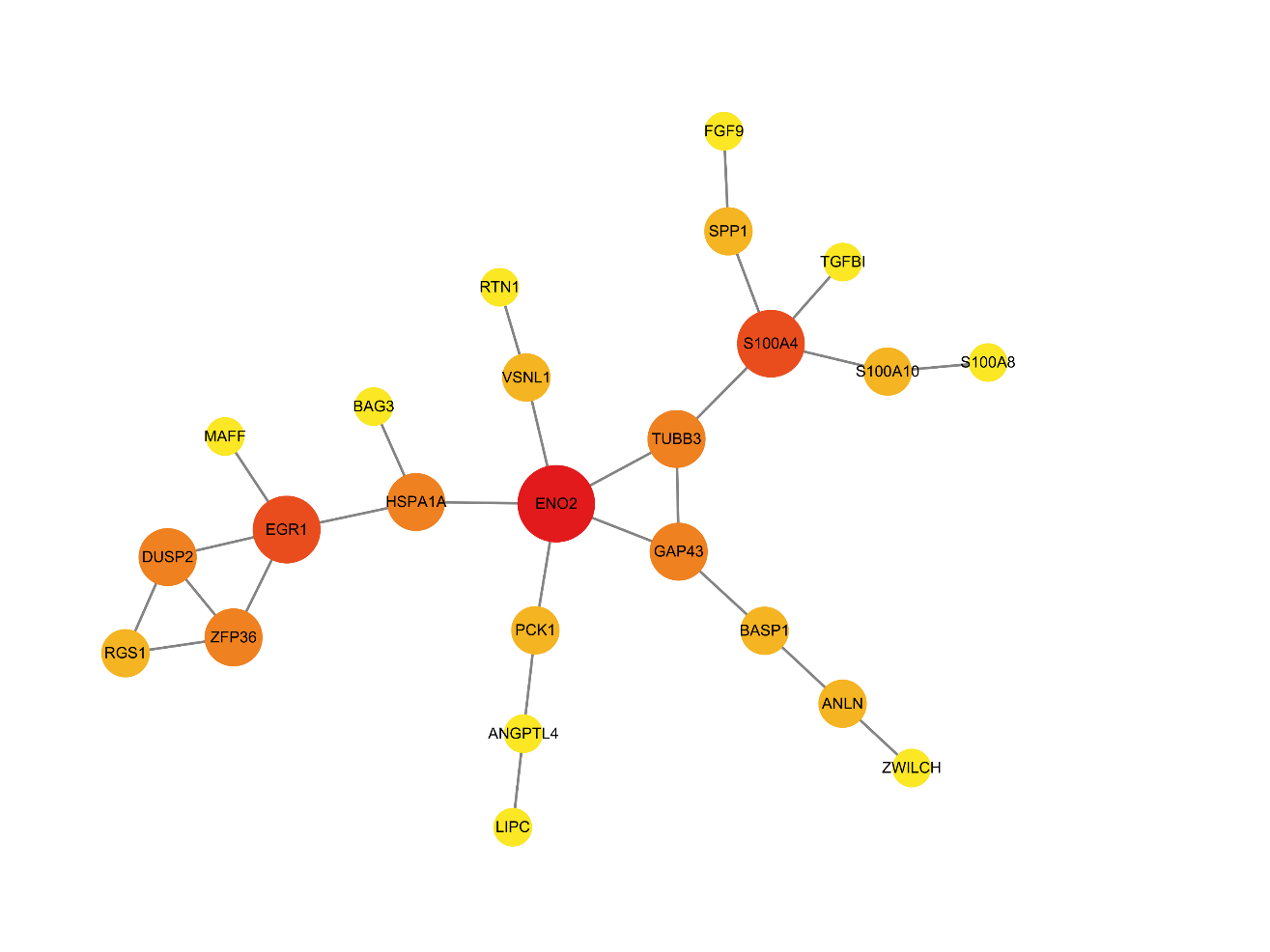


*Figure S4. Protein–protein interaction (PPI) analysis of shared DEGs.*

STRING-derived PPI network highlighting S100A4, ENO2, and EGR1 as key hub genes.


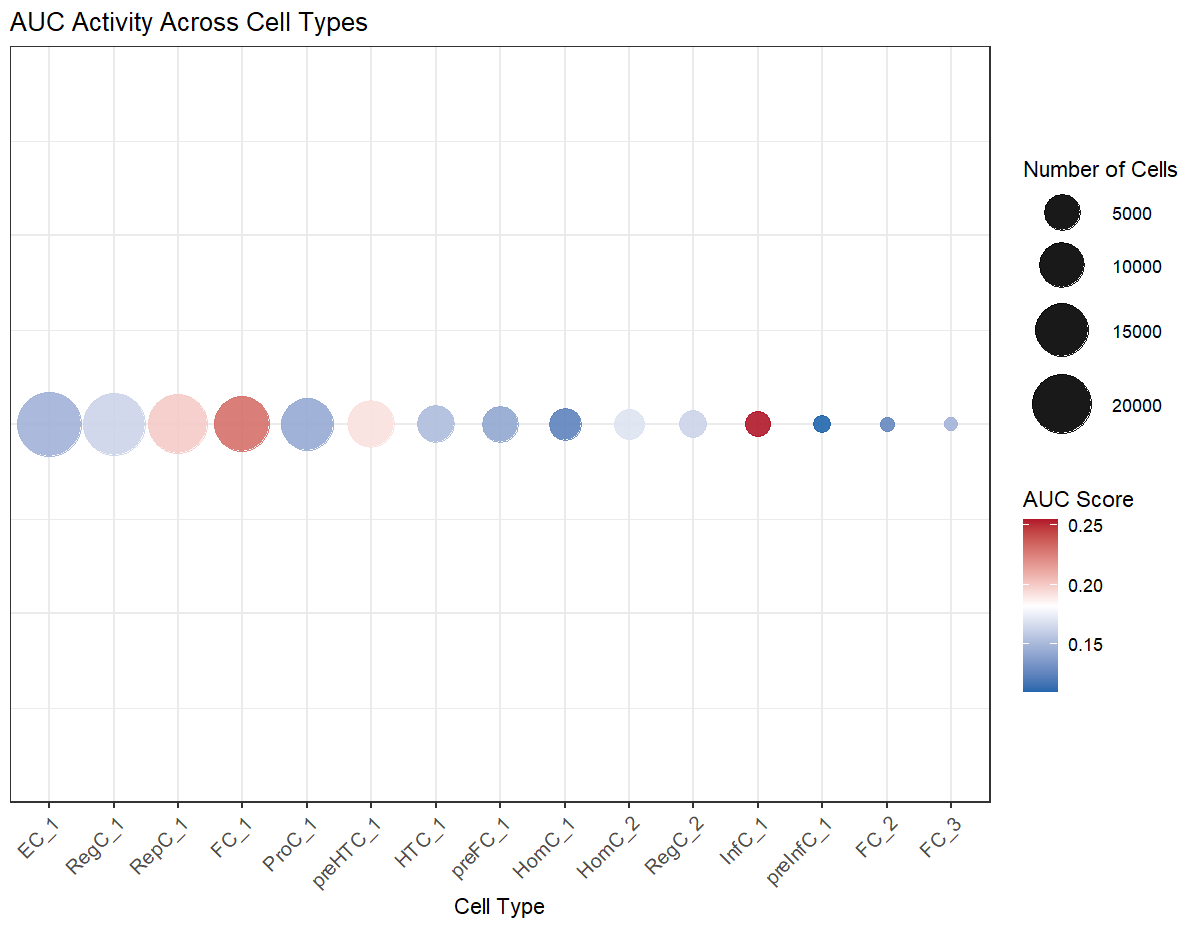


*Figure S5. SUGS activity (AUC score) across OA cell subpopulations*

The inflammatory chondrocyte subtypes (InfC_1), fibrochondrocytes (FC_1), and pre‑hypertrophic chondrocytes (preHTC_1) exhibited markedly elevated SUGS activity.


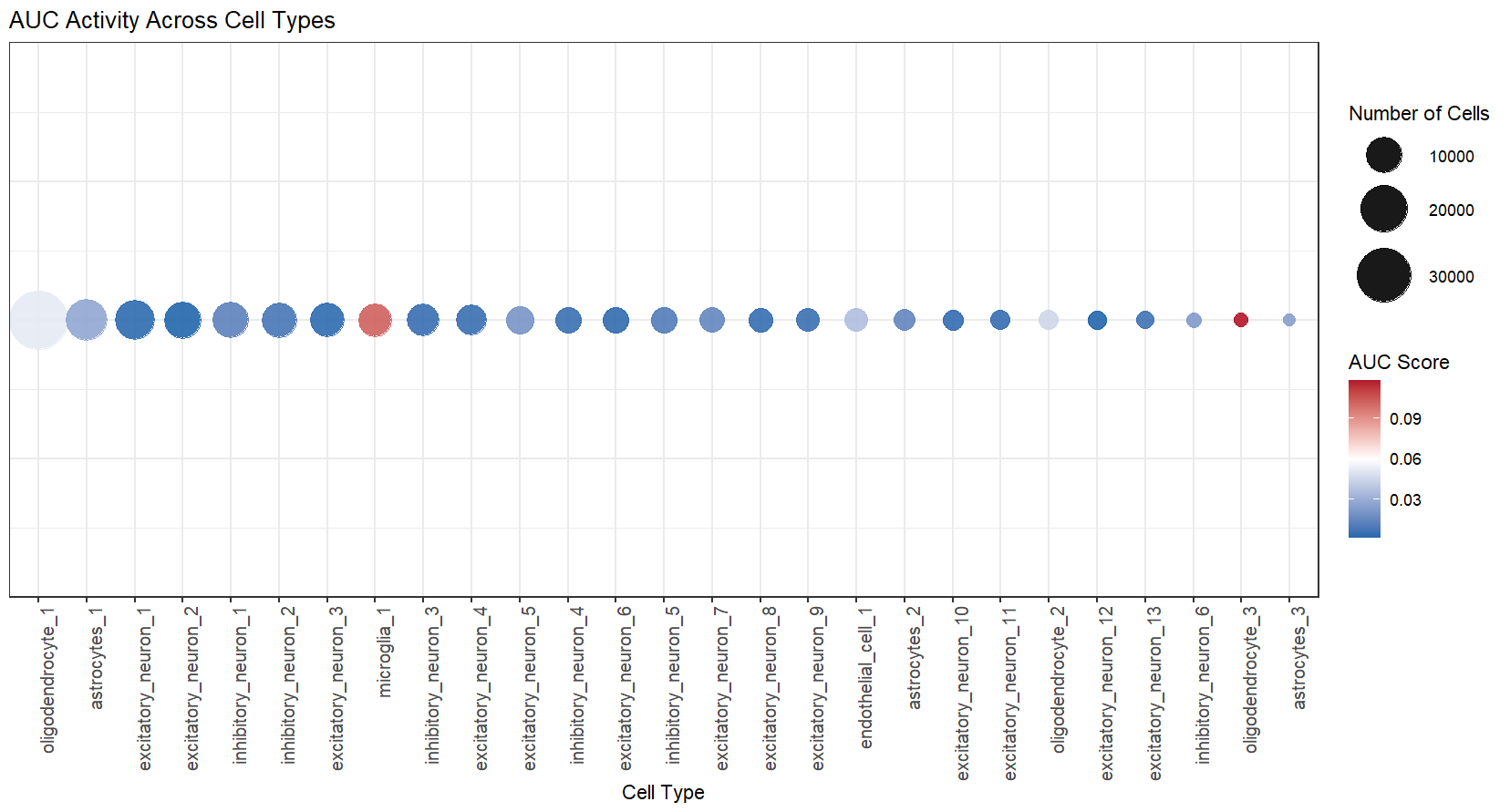


*Figure S6. SUGS activity (AUC score) across AD cell subpopulations*

The microglia_1 and oligodendrocyte_3 subpopulations exhibited higher SUGS activity. In addition, based on a combined assessment of AUC scores and cell abundances, the subpopulations oligodendrocyte_1, astrocytes_1, excitatory_neuron_5, and endothelial_cell_1 were selected for comparative analyses.


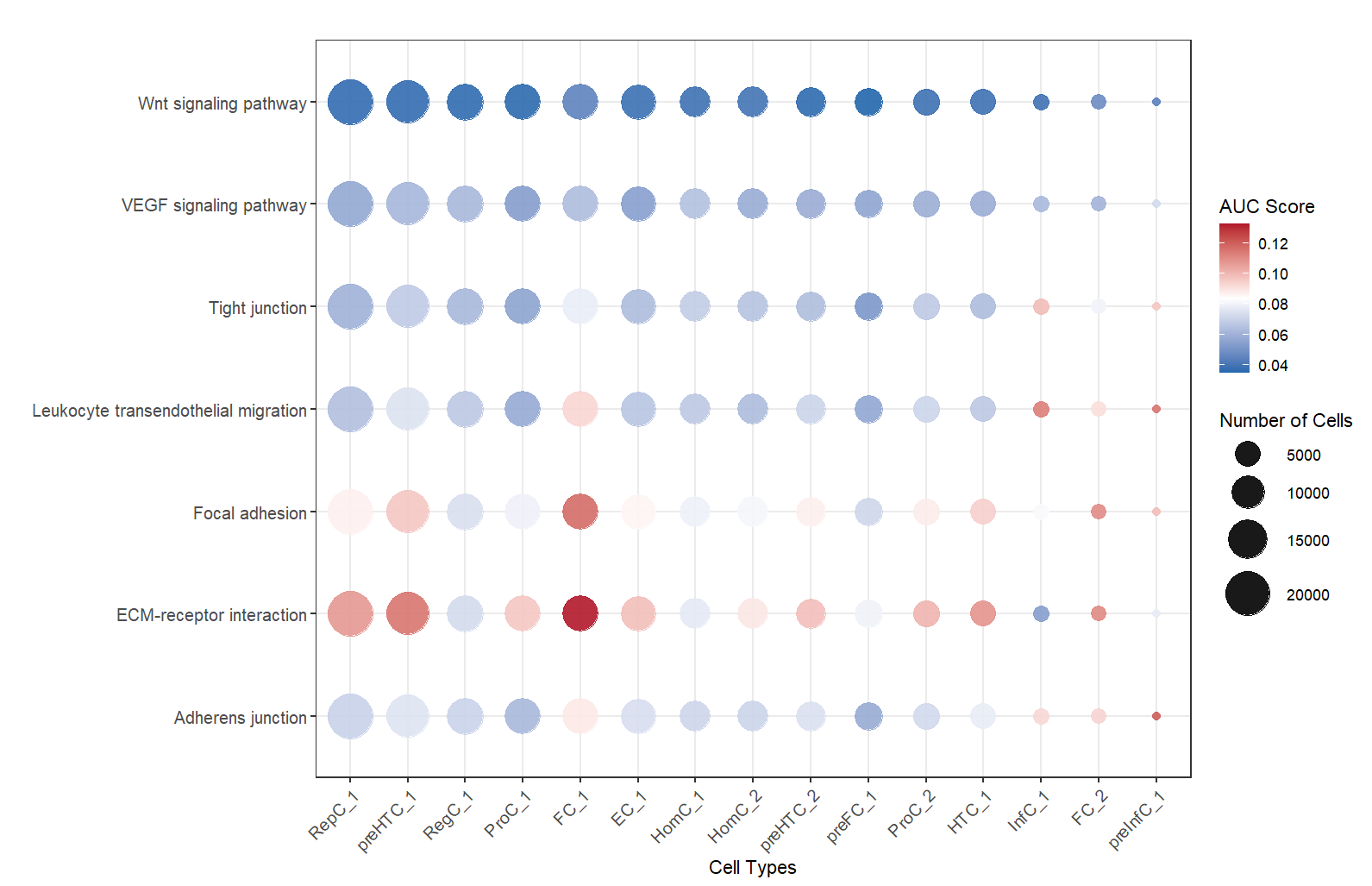


*Figure S7. Neurovascular- and Endothelial-Associated Pathway Activity across OA cell subpopulations*

ECM-receptor interaction and Focal adhesion showed higher pathway activity scores in the key disease-relevant subpopulations preHTC_1 and FC_1, while InfC_1 displayed elevated scores for pathway categories related to Tight junction, Adherens junction, and Leukocyte transendothelial migration.


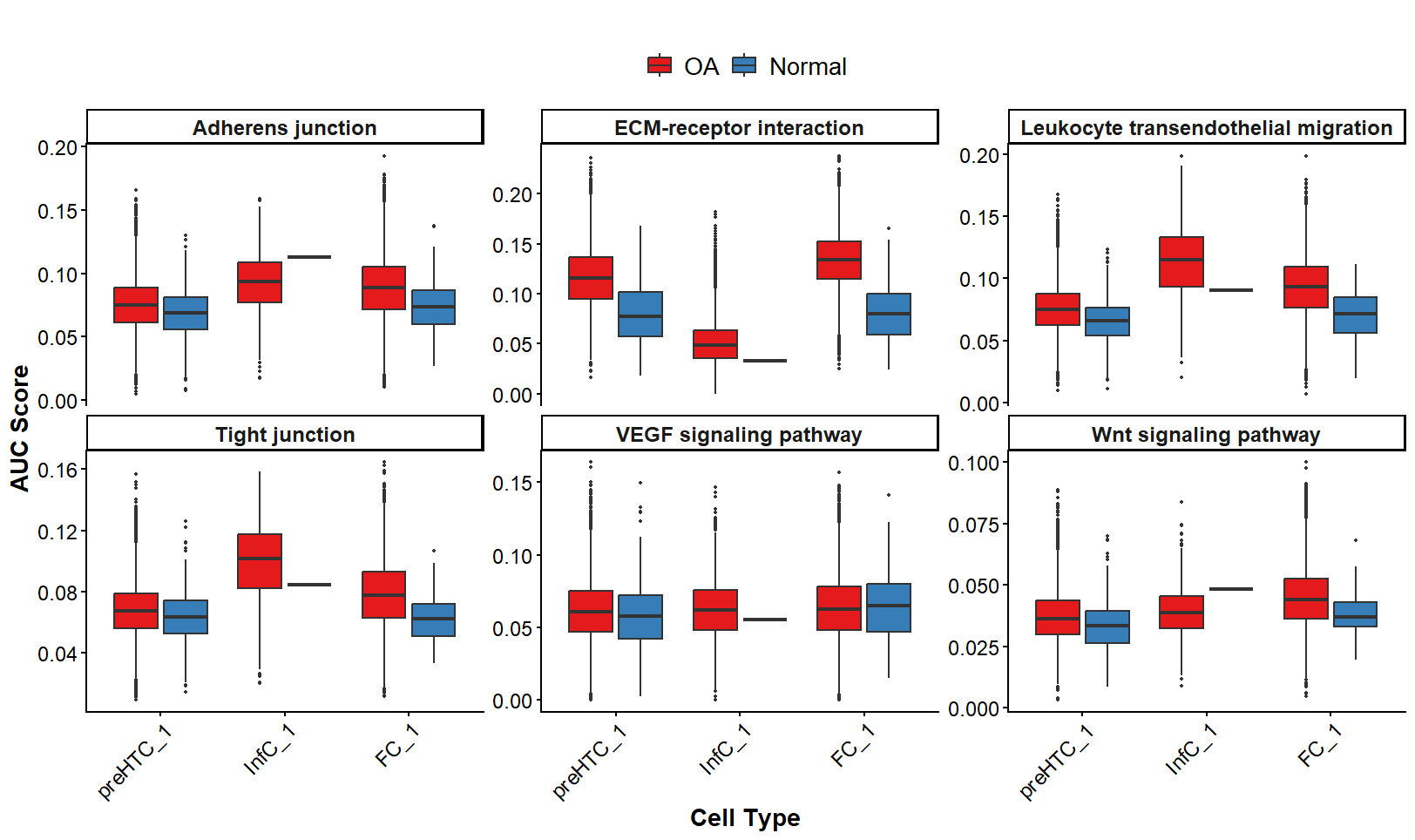


*Figure S8. Boxplots of Neurovascular- and Endothelial-Associated Pathway Activity in Specific OA Cell Subclusters*

AUCell-based scoring of Neurovascular- and Endothelial-Associated Pathway activity across individual OA (red) and normal (blue) cells.


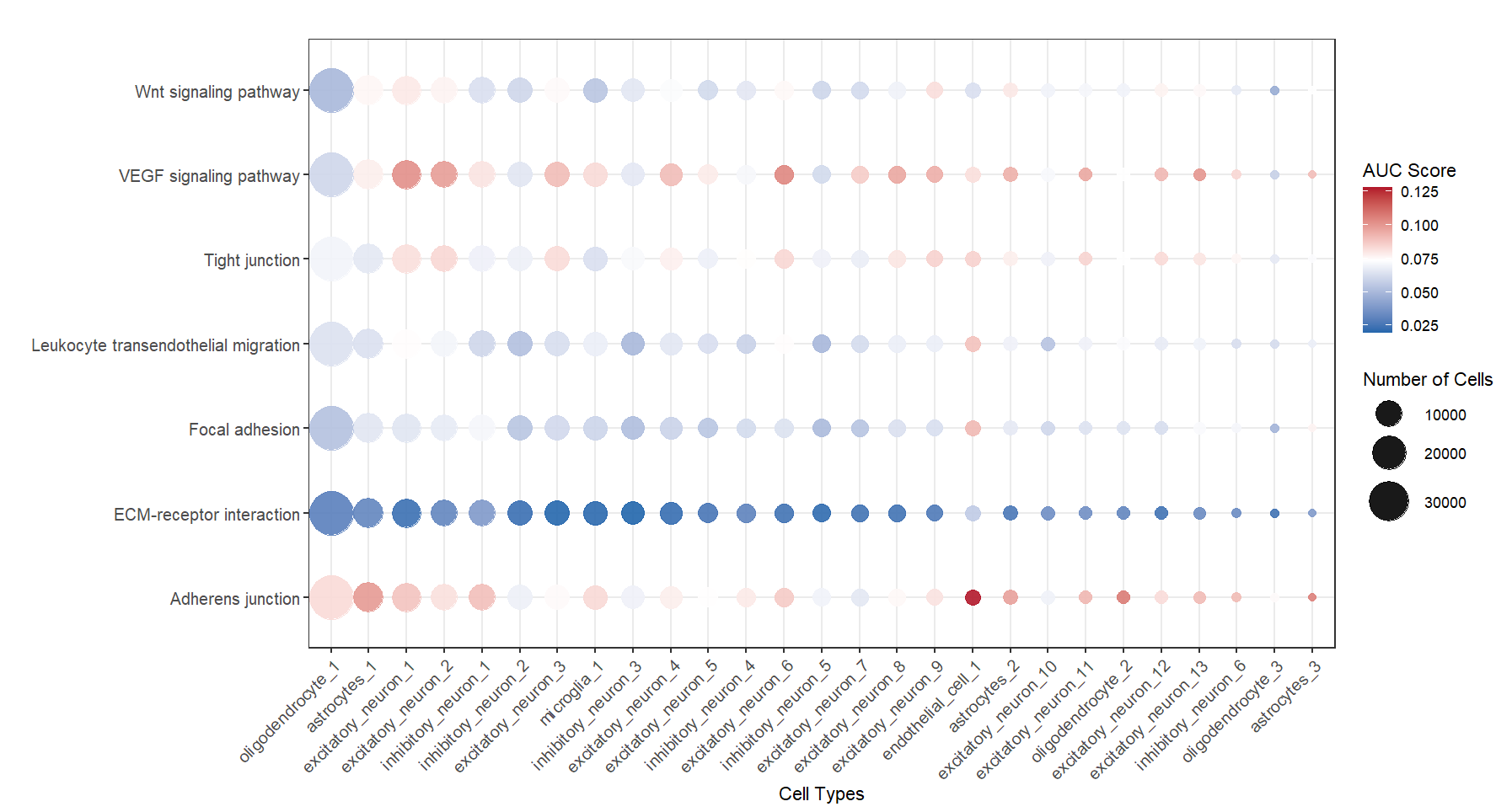


*Figure S9. Neurovascular- and Endothelial-Associated Pathway Activity across AD cell subpopulations*

In AD, astrocytes_1 and endothelial_cell_1 also showed signal across selected neurovascular- and endothelial-associated pathway categories, including Adherens junction, VEGF signaling, and Wnt signaling.


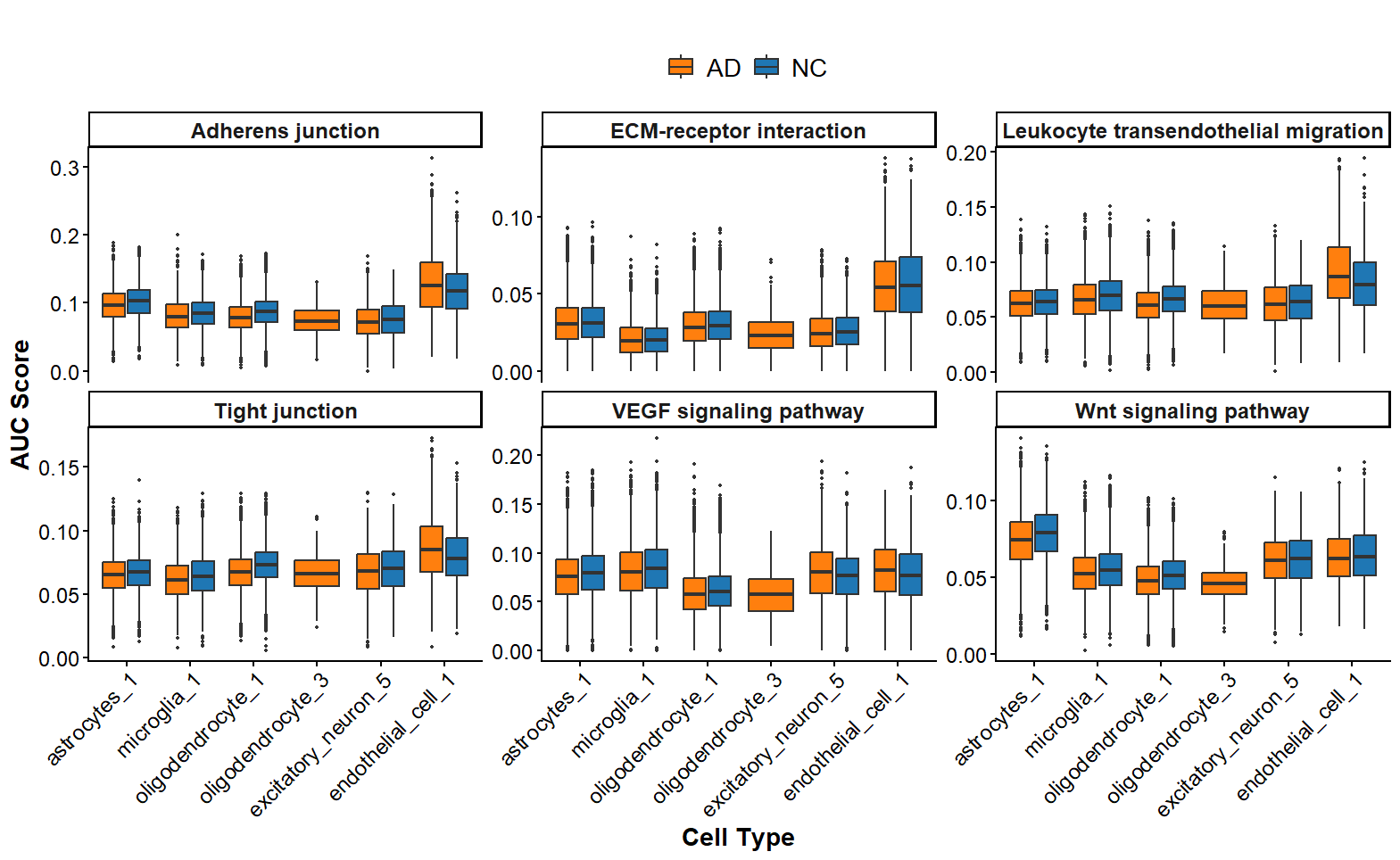


*Figure S10. Boxplots of Neurovascular- and Endothelial-Associated Pathway Activity in Specific AD Cell Subclusters*

AUCell-based scoring of Neurovascular- and Endothelial-Associated Pathway activity across individual AD (orange) and normal (blue) cells.
